# Ecological Drivers of Microbiota Diversity in the Pharmacophagous Turnip Sawfly

**DOI:** 10.1111/1462-2920.70309

**Published:** 2026-04-21

**Authors:** Pragya Singh, Philipp Dirksen, Martin Kaltenpoth, Caroline Müller

**Affiliations:** ^1^ Chemical Ecology Bielefeld University Bielefeld Germany; ^2^ Department of Insect Symbiosis Max Planck Institute for Chemical Ecology Jena Germany; ^3^ Evolutionary Ecology, Institute of Organismic and Molecular Evolution (iomE) Johannes Gutenberg University Mainz Germany; ^4^ Joint Institute for Individualisation in a Changing Environment (JICE) University of Münster and Bielefeld University Bielefeld Germany

**Keywords:** chemical ecology, holometabolous insect, host–microbiota interactions, nutritional stress, plant–insect–microbe interactions, sawfly

## Abstract

Animals encounter diverse ecological factors that shape their microbiota. While plants often provide nutrition, some animals also utilise them for non‐nutritional purposes, that is, pharmacophagy. Although both uses may influence herbivore microbiomes, the effects of pharmacophagy remain underexplored. We studied such effects in the holometabolous insect *Athalia rosae*, whose larvae feed on Brassicaceae plants, while adults take up nectar from Apiaceae and, additionally, clerodanoids (plant specialised metabolites) through pharmacophagy from Lamiaceae species. We examined how nutritional and non‐nutritional plant use affect microbiota diversity, composition and predicted function. We also tested whether the microbiota of larval faeces can serve as a non‐invasive proxy for larval microbiota and compared microbiota profiles across rearing environments (laboratory vs. wild) and specific life stages (i.e., larvae vs. adults). Microbiota composition was highly context‐dependent, with indicator species analyses revealing treatment‐specific amplicon sequence variants. Diet, starvation and clerodanoid access each resulted in distinct communities; faeces reliably reflected larval microbiota; and wild‐caught adults harboured the most diverse communities. Predicted microbial functions varied with diet and pharmacophagy, particularly in pathways putatively linked to metabolite degradation, energy metabolism and detoxification. Together, our findings suggest that both nutritional and non‐nutritional plant use shape intraspecific microbiota variation in insects.

## Graphical abstract

We show that both nutritional (host‐plant diet, starvation) and non‐nutritional (pharmacophagy) plant use independently shape microbiota diversity, composition and predicted function in *Athalia rosae*. Pharmacophagy via clerodanoid uptake altered microbial communities and enriched metabolic pathways linked to plant compound degradation. Wild individuals harboured the most diverse microbiota, and larval faeces reflected gut communities, providing a non‐invasive proxy for ecological microbiome studies.

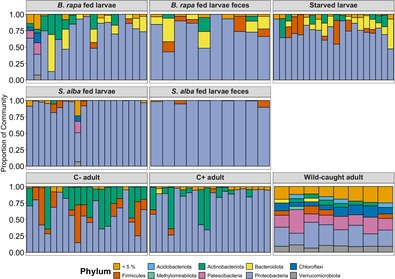

## 1 Introduction

Many animals harbour diverse microbial communities that profoundly influence their physiology and ecology (Douglas 2018). Within species, these microbial communities can vary across specific life stages and ecological conditions (Lange et al. 2023), and identifying the ecological drivers of this variation is important for understanding the adaptive significance of the host‐associated microbiota (Kolodny et al. 2020). In herbivorous insects, one such ecological driver is their association with plants, which can be multifaceted and form a key component of an individual's niche. While interactions between plants and herbivorous insects are often viewed as a nutritional relationship, insects can also utilise plants for specific non‐nutritional purposes. Such targeted uptake, sequestration and utilisation of plant compounds in insects for functions other than nutrition is termed pharmacophagy (Boppré 1984; Singh and Müller 2025). Different purposes of this non‐nutritional usage of plants can include chemical defence against predation, self‐medication or pheromone synthesis (Beran and Petschenka 2022; De Roode et al. 2013; Erler et al. 2024; Nishida 2014). Similar phenomena of non‐nutritional usage of plants have also been observed in non‐insect taxa (Pusceddu et al. 2025). For example, parasitised chimpanzees chew the bitter pith of *Vernonia amygdalina*, which is linked to alleviating parasite‐associated symptoms (Huffman et al. 1993). While dietary effects of different host plants on microbiota of herbivores have been well described (Hammer et al. 2017; Jose et al. 2023; Liu et al. 2020), impacts of pharmacophagy on herbivore microbiota remain understudied, despite the possible influence on host‐associated microbial diversity and function.

A potential mechanism through which plant association may shape herbivore microbiota is variation in plant chemistry, as plants differ in their composition of primary and specialised metabolites. Indeed, variation in phytochemicals may shape the host plant microbiota (Malacrinò et al. 2025), which could then influence associated insect microbial communities. In particular, plant metabolites with antimicrobial properties may modulate gut microbiota of herbivores by affecting microbial survival and composition (Šigutová et al. 2024). Moreover, plant quality and phytochemicals may also affect insect immunity, for example, nutritional stress or chemically challenging diets may reduce immune investment and thereby increase susceptibility to pathogenic microbial infection (Cory and Hoover 2006; Ode and Ghosh 2025). On the insect side, gut microbiota can contribute to metabolic pathways involved in the degradation of toxic plant metabolites and thus complement detoxification mechanisms and enable herbivory (Ceja‐Navarro et al. 2015; Dearing et al. 2022; Douglas 2015; Xia et al. 2017). Beneficial bacterial symbionts also support digestion, such as the breakdown of plant cell walls in herbivorous insects, thereby contributing to host fitness and ecological adaptation (Kaltenpoth et al. 2025; Kirsch et al. 2025). Thus, plant metabolites and herbivore microbiomes can be closely interlinked.

Beyond plant chemical composition, other ecological factors may shape microbiota variation. One such factor is food limitation. Animals often face periods of reduced food availability, leading to nutritional stress or starvation, which in turn can alter their microbiota (Dillon et al. 2010; Mack et al. 2018; Xia et al. 2014; Yang et al. 2021). Such microbiota shifts may influence resource utilisation efficiency, host resilience, or disease susceptibility (Engel and Moran 2013). Alongside these resource‐based drivers, the origin (e.g., laboratory‐reared versus wild‐caught) of an individual may influence its microbiota composition. Indeed, studies have shown that laboratory‐reared insects may harbour distinct communities compared to field‐collected individuals (Baños‐Quintana et al. 2024; Polidori et al. 2023; Tinker and Ottesen 2021; Waltmann et al. 2019), due to limited environmental microbial exposure and dietary uniformity in the laboratory. Thus, it is important to understand how laboratory rearing may modulate microbiota compared to natural populations as this may have implications for extrapolating findings from laboratory studies to ecological contexts (Cusick et al. 2021).

Apart from the above‐mentioned external environmental factors, internal factors, such as pronounced ontogenetic shifts, can lead to the realisation of distinct ecological niches across certain life stages, for example in holometabolous insects, where larvae and adults often show different feeding modes. These transitions can be associated with shifts in microbiota composition (González‐Serrano et al. 2020; Hammer and Moran 2019; Lange et al. 2023; Manthey et al. 2023). As the insect microbiota can change over time and with ageing (Dillon et al. 2010), longitudinal studies are essential to capture such temporal dynamics (Gillingham et al. 2025). For such studies, non‐invasive methods, particularly faecal sampling, offer a promising way to monitor microbial communities without sacrificing hosts, enabling repeated sampling (Fink et al. 2013). However, the reliability of faecal samples as proxies for internal microbiota, especially under varying dietary conditions, needs to be established across species.

An excellent model system to examine microbiota variation across multiple ecologically relevant conditions is the turnip sawfly *Athalia rosae* (Hymenoptera: Tenthredinidae). This holometabolous species exhibits several traits that make it particularly suitable for investigating microbiota dynamics. Larvae of *A. rosae* feed on various Brassicaceae species (Travers‐Martin and Müller 2008), allowing us to test for potential microbial shifts in dependence on the host plant. Larval feeding can lead to defoliation, resulting in short‐term starvation, which has been shown to affect larval phenotypes in diverse ways (Brueggemann, Singh, and Müller 2025; Paul et al. 2022). Here, we examined whether such nutritional stress alters microbial diversity. Moreover, we evaluated whether the microbiota of larval faeces can serve as a proxy for larval microbiota, providing a non‐invasive tool for longitudinal sampling. Adult *A. rosae* feed on nectar of Apiaceae but additionally engage in pharmacophagy and acquire neo‐clerodane diterpenoids (hereafter “clerodanoids”) from Lamiaceae plants (Brueggemann et al. 2023). These non‐nutritional plant metabolites are used by the adults as chemical defences and enhance mating success and social interactions (Amano et al. 1999; Paul and Müller 2021; Singh et al. 2022, 2024). Since clerodanoids are also known for their antimicrobial properties (Bozov et al. 2015), we assessed whether clerodanoid access is associated with shifts in microbiota composition. We also compared microbiota profiles of laboratory‐reared and wild‐caught adults to explore the influence of rearing environment. To identify microbial taxa driving these compositional differences across treatments, we applied indicator species analyses to detect amplicon sequence variants (ASVs) consistently associated with specific ecological contexts. Finally, we assessed how the functional profile of the *A. rosae* microbiota varies with larval diet and adult pharmacophagy.

## 2 Materials and Methods

### 2.1 Sample Collection and Experimental Design

Individuals of *A. rosae* , except for wild‐caught adults, were obtained from a laboratory stock population originally established from sawflies collected in and around Bielefeld, Germany. To maintain genetic diversity, the population was supplemented annually with field‐caught individuals (see Singh et al. 2023 for rearing details). The laboratory sawfly population was maintained in mesh cages (60 × 60 × 60 cm) at 15°C–25°C, 60% relative humidity, and a 16:8 h light:dark cycle. Bugleweed ( *Ajuga reptans* , Lamiaceae) and cabbage ( *Brassica rapa* var. *pekinensis*, Brassicaceae) were cultivated in a greenhouse, while white mustard ( *Sinapis alba* , Brassicaceae) was grown in a climate chamber. All plants were maintained at ≥ 20°C, with a 16:8 h light:dark cycle and 70% relative humidity. To standardise experimental conditions and reduce potential confounding variation associated with sex, as found, for example, in the microbiota of fruit flies (Aoki et al. 2025), only male sawflies were used (except for wild‐caught adults). To obtain male larvae, unmated females were allowed to oviposit on *S. alba* , generating male offspring via unfertilised eggs, as *A. rosae* is haplodiploid. Freshly hatched larvae were individually reared in Petri dishes and assigned to one of three treatments to examine how host plant and nutritional stress influence the microbiota. Larvae in the ad libitum feeding groups were reared on either *B. rapa* (*n* = 20) or *S. alba* (*n* = 20) leaf discs (~2.5 cm in diameter), with fresh leaf discs provided daily. In the starvation treatment (*n* = 20), larvae were fed with *B. rapa* until reaching the fourth instar, after which they were starved for 1 day directly post‐moult. Additionally, to examine microbiota overlap between larvae and their food source, leaf samples were collected from *S. alba* (*n* = 5) and *B. rapa* (*n* = 5) plants from which feeding discs had been cut. One day after moulting to the fourth instar, all larvae were flash‐frozen in liquid nitrogen and stored at −80°C. Faecal samples were collected from randomly chosen *B. rapa* ‐fed (*n* = 10) and *S. alba* ‐fed (*n* = 10) larvae before flash‐freezing.

**FIGURE 1:**
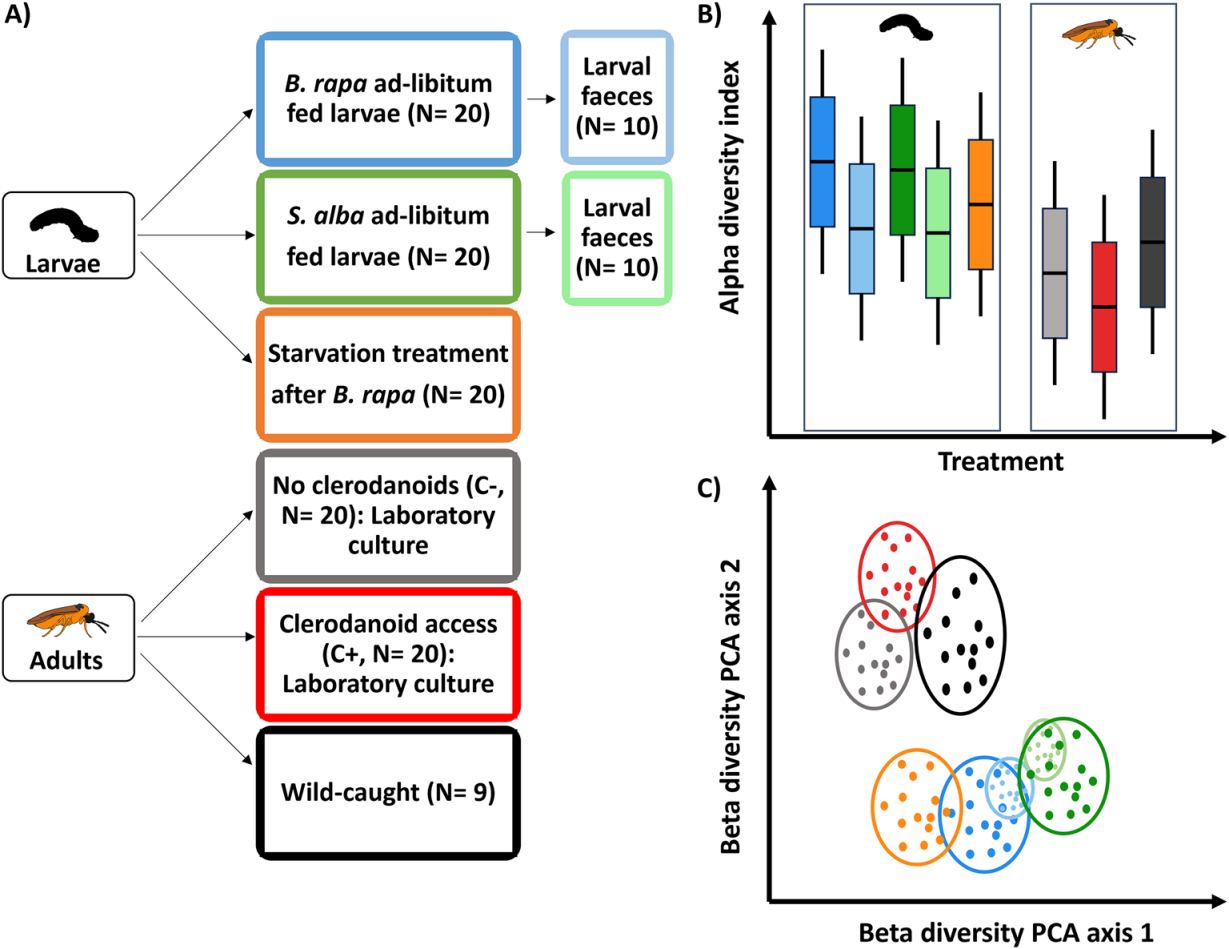
(A) Experimental design to examine microbiota diversity and composition in *Athalia rosae* across larval diet ( *Brassica rapa* vs. *Sinapis alba* ), larval nutritional stress (starvation vs. ad libitum feeding), adult non‐nutritional uptake of plant clerodanoids (C− vs. C+), rearing conditions (laboratory‐reared vs. wild‐caught adults) and specific life stages (larvae vs. adults). Faecal samples were collected from larvae to assess their suitability as proxies for larval microbiota. Note that all individuals used were males, except for wild‐caught adults (six females, three males). (B) We hypothesised that the larvae would have higher microbial alpha diversity than adults and that C− adults would have lower alpha diversity than C+ adults. (C) We expected the adult microbiota composition to cluster separately from that of the larvae. We expected the larvae to cluster depending on their diet and the larval faeces to cluster with the larvae from the same diet.

Freshly eclosed males from the rearing culture were assigned to one of two treatment groups to assess the effect of clerodanoid uptake on the microbiota. Individuals in the clerodanoid access (C+) group (*n* = 20) were provided with a ~1 cm^2^ leaf of *A. reptans* , while individuals in the control group (*n* = 20) had no access to clerodanoids (C−). Adult *A. rosae* lack chewing–biting mouthparts and can only take up nectar or other compounds by licking; thus, clerodanoids are acquired via pharmacophagy through interaction with the leaf surface, such as by licking (Brueggemann, Fleer, and Müller 2025). All adults had access to a 2% (v/v) honey: water solution. After 48 h, individuals were flash‐frozen in liquid nitrogen and stored at −80°C. Wild *A. rosae* adults (*n* = 9; six females and three males) were collected near Bielefeld (52° 02′ 41.7″ N, 8° 29′ 46.7″ E) in September 2022. Individuals were transported to the laboratory in Falcon tubes (50 mL), flash‐frozen in Eppendorf tubes using liquid nitrogen, and stored at −80°C until further processing. For an overview of the experimental setup, see Figure 1.

### 2.2 DNA Extraction, Amplification and Sequencing

Microbial DNA was extracted with ZymoBIOMICS DNA Miniprep Kit (Zymo Research) using a protocol optimised for these samples (see Supporting Information S1 for details, Figure S1). We included negative controls (*n* = 9) during the extraction process, that were spread through the different extraction batches. Sequencing of the 16S rRNA gene was outsourced to StarSEQ GmbH (Mainz, Germany). The V3–V4 region of the bacterial 16S rRNA gene was amplified using the primers 341 F (5′‐CCTACGGGNGGCWGCAG‐3′) and 805R (5′‐GACTACNVGGGTWTCTAATCC‐3′) (Klindworth et al. 2013). PCR amplification included positive (*n* = 2, ZymoBIOMICS Microbial Community DNA Standard D6305/D6306) and negative (*n* = 2) controls provided by the sequencing facility. Amplicons were sequenced on an Illumina MiSeq platform (2 × 300 bp) following the manufacturer's protocol.

### 2.3 Quantitative PCR for Bacterial Abundance Assessment

Total bacterial abundance was assessed by quantitative PCR (qPCR) to estimate bacterial 16S rRNA copies as a proxy. Reactions (20 μL) contained 1 μL template DNA, 0.8 μL each of the forward and reverse primers (fwd: EUB338mod, TCCTACGGGAGGCAGCAG; rev: EUB518, ATTACCGCGGCTGCTGG), 10 μL qPCR SYBR Green Mix (Biozym Blue S'Green qPCR Kit Separate ROX), and 7.4 μL water. The reactions were run on a CFX Connect Real‐Time PCR Detection System (Bio‐Rad, Hercules, CA, USA) using the following programme: initial denaturation at 95°C for 3 min, 40 cycles of denaturation at 95°C for 15 s and annealing/extension at 60°C for 30 s, followed by a final denaturation step at 95°C for 10 s. Then a melting curve was recorded by increasing the temperature by 0.5°C every 5 s from 55°C to 95°C, in order to assess amplification specificity. An eight‐point 10‐fold standard dilution series (10^−1^ to 10^−8^ ng μL^−1^) was used to generate standard curves, which were highly linear (*R* ^2^ ≈0.99). Starting quantity (SQ) values were calculated by interpolating sample quantification cycle (Cq) values against the corresponding standard curve and were used as estimates of the initial amount of bacterial 16S rRNA gene template in each sample.

### 2.4 Bioinformatics Pipeline

To detect and trim primers, demultiplexed Illumina sequence data were initially processed with Cutadapt (v4.2) (Martin 2011). Subsequently, the primer‐trimmed sequences were imported into QIIME2 (Quantitative Insights into Microbial Ecology 2, v2024.10) (Bolyen et al. 2019). The read quality was assessed by examining quality plots in QIIME2. Low‐quality bases were removed and Amplicon Sequence Variants (ASVs) were inferred using the q2‐dada2 (plugin) implementation of the DADA2 (Divisive Amplicon Denoising Algorithm) (Callahan et al. 2016), with truncation of forward and reverse reads at 280 and 220 base pairs, respectively. Taxonomic classification of ASVs was performed using a naive Bayes classifier trained on the SILVA SSU 138 reference database (99% OTUs) with q2‐feature‐classifier (Quast et al. 2012). We extracted reference sequences specific to this region using our primer sequences and these corresponding target sequence fragments were used to train the classifier. The data was then imported into R and using the ‘prevalence’ method (probability threshold = 0.1, package ‘decontam’ ver. 1.22.0, Davis et al. 2018), the sequence contaminants were identified and removed. Next the data were imported back into QIIME2. To exclude non‐bacterial taxa, ASVs classified as mitochondria, chloroplasts, Archaea, Vertebrata, Eukaryota or unassigned were filtered out using the q2‐taxa filter‐table plugin, followed by the removal of singletons using q2‐feature‐table filter‐features plugin. The remaining ASVs were aligned using MAFFT (Katoh 2002) with the q2‐alignment plugin, and the aligned sequences were used to construct a phylogeny with FastTree (Price et al. 2010) with the q2‐phylogeny plugin. To evaluate sequencing depth and sample coverage, rarefaction curves were then generated at a maximum depth of 5000 with q2‐diversity‐alpha‐rarefaction plugin (Figure S2). Samples with fewer than 1000 sequencing reads were excluded from downstream analyses, resulting in the removal of 12 samples: five *B. rapa* leaf samples, three *S. alba* leaf samples, three larvae reared on *B. rapa* and one wild‐caught adult. As only two *S. alba* leaf samples remained, these were also excluded from downstream analysis (but see Figure S3).

## 3 Statistical Analyses

All data visualisation and analysis were conducted using R v 4.4.2 (R Core Team 2024), unless otherwise mentioned. Differences in total bacterial abundance among treatments were examined using Kruskal–Wallis rank‐sum tests, followed by post hoc Dunn tests with Benjamini‐Hochberg correction. Microbial alpha diversity was quantified with Shannon entropy (Shannon 1948) and Faith's Phylogenetic Diversity (Faith's PD) (Faith 1992), calculated via the core‐metrics‐phylogenetic method (q2‐diversity plugin) with rarefaction to 1500 sequences per sample. This sequencing depth was chosen as a conservative threshold, as rarefaction curves approached saturation below this level (Figure S2), while maximising sample retention. We examined the differences in alpha diversity metrics using Kruskal–Wallis rank‐sum tests, followed by post hoc Dunn tests with the Benjamini‐Hochberg correction.

The relative abundance of microbial taxa at the phylum level for each sample was calculated. Note that microbial taxa are reported based on the nomenclature used in the SILVA 138 reference database. Although some phylum names have been revised in recent taxonomic frameworks, we retained the SILVA‐based terminology throughout for consistency with our classification pipeline and with common usage in the 16S rRNA gene literature. We also identified treatment‐specific core microbiota (microbiome package, Lahti and Shetty 2018) with a minimum prevalence of 70% and minimum relative abundance of 1% across the samples of a treatment. We additionally evaluated the robustness of core sizes across detection thresholds (0.1%, 0.5%, 1%, 2% relative abundance) and prevalence thresholds (50%, 70%, 90%).

We examined microbial beta diversity using Aitchison distance (Aitchison et al. 2000) to accommodate the compositional nature of microbiota data (Gloor et al. 2017). To compute this, we first applied a centred log‐ratio (CLR) transformation to the ASV table using the transform function in the microbiome R package. To account for zero values prior to CLR transformation, a small pseudocount equal to half the minimum non‐zero value in the dataset was added, following the default behaviour of the microbiome R package. We then calculated Euclidean distances on the CLR‐transformed data, which corresponds to Aitchison distance, and conducted a principal component analysis (PCA) to visualise differences in microbial community composition. Differences in beta diversity among treatments were tested using PERMANOVA (adonis2 function, vegan package v2.6‐10, Oksanen et al. 2025). Pairwise PERMANOVA comparisons were conducted using the pairwise.adonis2 function from the pairwiseAdonis package to identify specific group‐level differences. To assess the homogeneity of group dispersions, we used the betadisper and permutest functions (999 permutations) from the vegan package, followed by a Tukey HSD post hoc test to identify specific group‐wise differences in dispersion. Indicator species (ASV) analysis was conducted using the *indicspecies* R package (Cáceres and Legendre 2009) to identify microbial ASVs that were consistently and preferentially associated with specific treatments. Analyses were performed on contrasts for larval diet (larvae fed ad libitum on *B. rapa* vs. *S. alba* ), larval feeding status (ad libitum‐fed on *B. rapa* versus starved larvae), adult treatment (C+ vs. C−), adult rearing origin (lab‐reared vs. wild‐caught) and developmental stage (all larvae vs. all adults). Before indicator analysis, ASVs occurring in fewer than 30% of samples within all treatment groups were excluded to reduce the influence of very rare taxa. Associations were quantified using the group‐equalised indicator value statistic (IndVal.g), with statistical significance assessed via permutation testing (999 permutations). To focus on the most diagnostic taxa, we summarised ASVs showing high indicator values (IndVal.g ≥ 0.7; permutation *p* ≤ 0.05) in the results, while full results for all significant ASVs are provided in the Supporting Information.

As shifts in microbial community composition may not necessarily translate into changes in overall microbiome function due to functional redundancy among taxa, we next assessed variation in predicted functional pathway abundances. We examined potential metabolic adaptations of the microbiota in larval and adult *A. rosae* using predictive functional profiling with PICRUSt2 (v2.6.2) (Douglas et al. 2020). To do this, we predicted MetaCyc metabolic pathways of the microbiota using the default pipeline ‘picrust2_pipeline.py’ and default parameters. Subsequently, the ‘add_descriptions.py’ was run to add the description of the pathways. Note that as this approach relies on 16S rRNA gene profiles, the resulting predictions should be interpreted as indicative of functional capacity and represent potential rather than actual microbial activity.

To identify metabolic pathways differing in abundance between treatment groups, we used Analysis of Compositions of Microbiomes with Bias Correction (ANCOM‐BC; ANCOMBC package v2.4.0; Lin and Peddada 2020). Differential abundance was assessed using a conservative Bonferroni‐adjusted *p* value threshold of ≤ 0.001 and an absolute log fold change (LFC) ≥ 2; all other parameters were left at default values. We focused on three pairwise comparisons: (1) *B. rapa* ‐fed larvae versus starved larvae, (2) *B. rapa* ‐fed larvae versus *S. alba‐*fed larvae and (3) adults without clerodanoid access (C−) versus those with access (C+).

## 4 Results

### 4.1 Bacterial Communities Associated With Wild‐Caught Adult Sawflies Exhibit Highest Alpha Diversity

Overall, the estimated total bacterial abundance as measured by qPCR did not differ significantly among the larval and larval‐faeces treatment groups (Figure S4). Similarly, the adult treatment groups did not differ significantly from one another. However, C+ and wild‐caught adults had significantly lower estimated bacterial abundance than the larval and larval‐faeces treatment groups.

**FIGURE 2:**
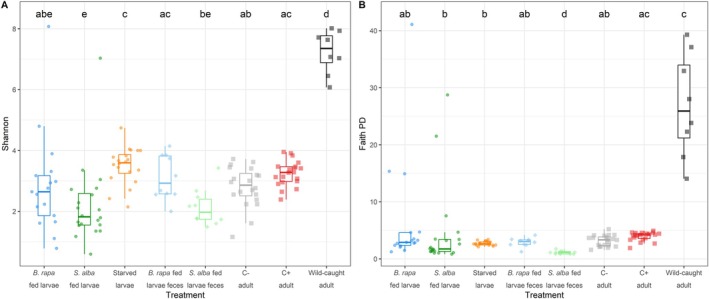
Alpha diversity indices of microbial communities across treatments in *Athalia rosae*. (A) Shannon entropy and (B) Faith's phylogenetic diversity (Faith's PD) for larval, faecal and adult samples. Boxplots display the median, interquartile range and individual sample points. Treatments included larvae fed ad libitum on *Brassica rapa* or *Sinapis alba* , starved larvae, faecal samples from larvae on both diets, laboratory‐reared adults unexposed (C−) or exposed to clerodanoids (C+) and wild‐caught adults.

To investigate how ecological context shapes microbiota diversity in *A. rosae* , we compared alpha diversity of microbes across specific insect life stages, diets, nutritional conditions, plant non‐nutritional metabolites and rearing environments. Overall, Shannon diversity varied significantly across treatments (Kruskal–Wallis *χ* ^2^ = 56.639, df = 7, *p* < 0.001; Figure 2A; Table S1), highlighting the context‐dependence of microbiota diversity. Larval diet had no effect on diversity, with individuals fed on *B. rapa* and *S. alba* showing comparable Shannon diversity. However, faecal samples from *B. rapa* ‐fed larvae exhibited higher diversity than those from *S. alba* ‐fed larvae (*p* = 0.032). Within diets, larval and faecal samples did not differ significantly in microbiota Shannon diversity, indicating that faeces provide a reliable proxy for Shannon diversity under controlled conditions. Starvation produced a notable shift, with starved larvae having significantly higher microbial Shannon diversity than microbiota of both ad libitum fed groups and faeces of *S. alba‐*fed larvae (all *p* ≤ 0.011), contrary to the expectation that nutrient limitation would reduce microbial diversity. This increase may reflect the proliferation of stress‐tolerant taxa under altered gut conditions. Finally, wild‐caught adults exhibited the highest microbial diversity overall, exceeding all laboratory‐reared groups (*p* < 0.05), likely reflecting the broader environmental exposure experienced in natural habitats.

Patterns based on Faith's PD were mostly consistent but revealed some important differences (*χ* ^2^ = 56.992, df = 7, *p* < 0.001, Figure 2; Table S2). While there were no differences in microbial Faith's PD between *B. rapa* ‐ and *S. alba* ‐fed larvae, *S. alba* ‐fed larvae exhibited significantly higher PD than their faeces (*p* = 0.020). In contrast to the Shannon‐based increase, starved larvae did not differ significantly from the fed larval groups or *B. rapa* faeces in Faith's PD, except for a single comparison with *S. alba* faeces (*p* = 0.005), indicating that the increase in richness under starvation did not correspond to greater phylogenetic breadth. Wild‐caught adults also had significantly higher microbial PD than all other treatments (*p* < 0.05) except C+ adults, reinforcing the influence of rearing environment on microbiota diversity.

### 4.2 Both Host Plant Diet and Non‐Nutritional Plant Metabolites Influence Microbial Community Composition

**FIGURE 3:**
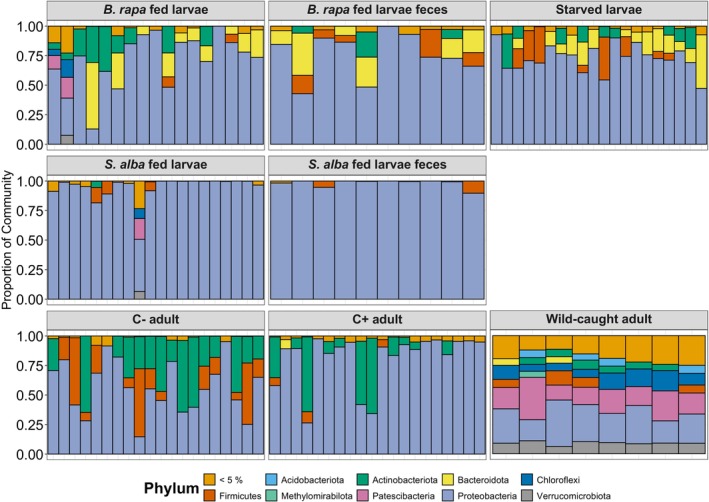
Relative abundance of bacterial phyla across treatments in *Athalia rosae*, shown as stacked bar plots. Each bar represents an individual sample, and panels indicate distinct experimental treatments: larvae fed ad libitum on *Brassica rapa* or *Sinapis alba* , starved larvae, faecal samples from larvae on both diets, laboratory‐reared adults unexposed (C−) or exposed to clerodanoids (C+) and wild‐caught adults. In wild‐caught adults, the first three bars represent males, while the remaining five correspond to female replicates. Only phyla with ≥ 5% relative abundance are shown.

Microbial community composition was dominated by Proteobacteria across most groups, particularly in larvae and lab‐reared adults (Figure 3, see also Figures S5 and S6, Table S3A,B). Using a prevalence ≥ 70% and a relative abundance detection threshold of ≥ 1%, the treatment‐specific core microbiota was small, ranging from 0 to 5 ASVs across groups. The number of core microbiota ASVs was highest in C+ adults (5 ASVs), intermediate in larval and faecal treatments (1–3 ASVs), and there were no core microbiota in wild‐caught adults (0 ASVs) (Figure S7, Table S4). The number of core taxa declined with increasing detection thresholds and higher prevalence requirements (Figure S8), such that larger ‘core’ sets appeared only under more permissive settings (notably for wild‐caught adults at low detection thresholds).

**FIGURE 4:**
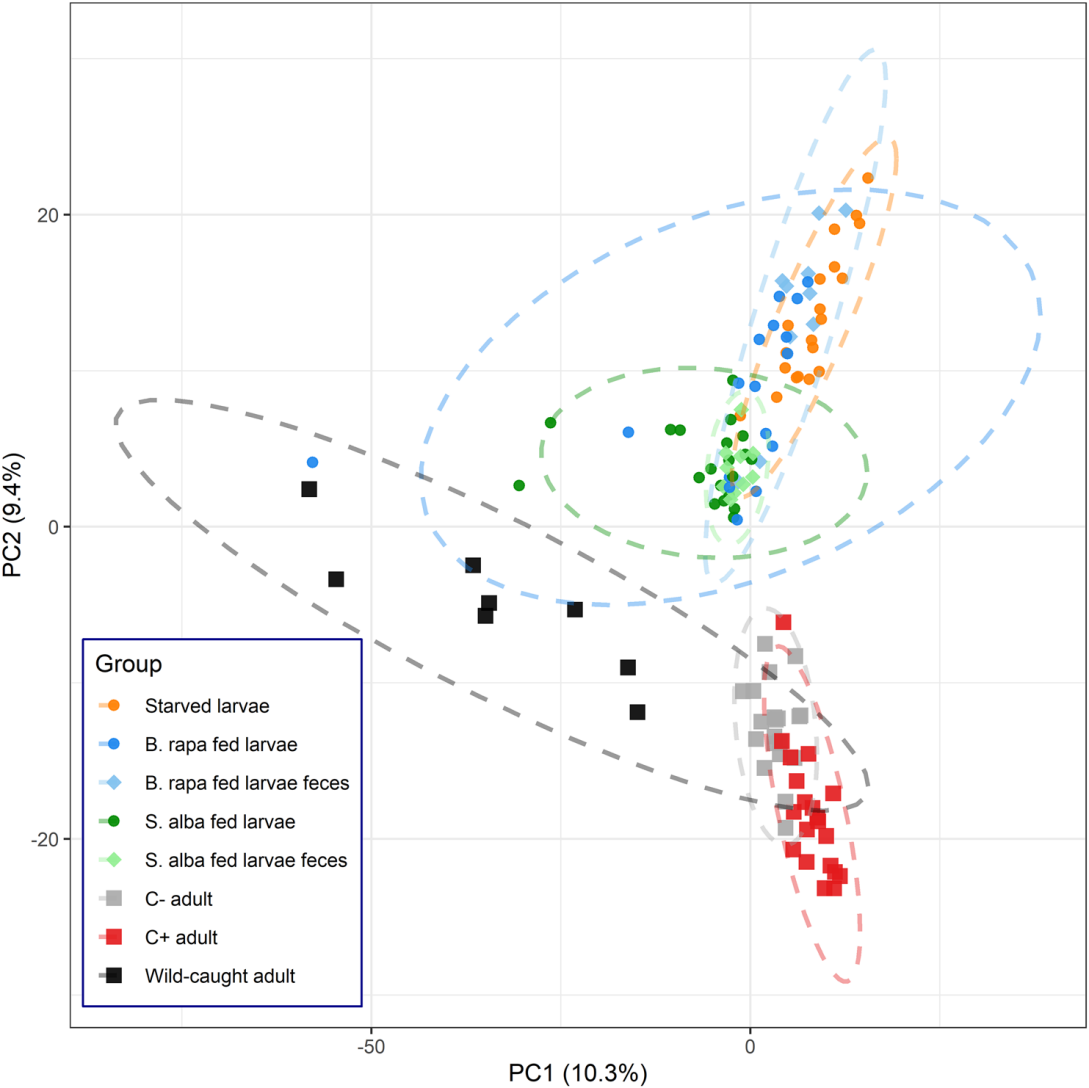
Principal component analysis (PCA) of CLR‐transformed amplicon sequence variant (ASV) data, based on Aitchison distance, illustrating variation in microbial community composition across *Athalia rosae* treatments. Each point represents an individual sample, coloured and shaped by treatment group. Groups include larvae subjected to different dietary treatments ( *Brassica rapa* vs. *Sinapis alba* ), corresponding larval faecal samples, starved larvae, adults unexposed (C−) or exposed to clerodanoids (C+), and wild‐caught adults. Ellipses denote 95% confidence intervals for group centroids. The percentages on each axis represent the variance explained by the first two principal components.

PCA revealed distinct microbial community clustering by life stage and rearing: larvae and their faeces formed separate clusters from adults, and wild‐caught adults clearly separated from lab‐reared ones (Figure 4). Microbial composition differed significantly across treatments (PERMANOVA; *R* ^2^ = 0.24, *F*_7,117_ = 5.35, *p* = 0.001), although multivariate dispersion was unequal (PERMDISP: *F*_7,117_ = 22.20, *p* = 0.001). Post hoc tests revealed that this difference was mainly driven by wild‐caught adults (*p* < 0.001; Table S5, Figure S9), with all other groups showing similar dispersion except one other comparison (microbiota of *B. rapa* ‐fed larvae vs. microbiota of faeces from *S. alba* ‐fed larvae: *p* = 0.005).

Pairwise PERMANOVA revealed significant differences in microbial community between most treatment groups (*p* < 0.05 for 26 of 28 comparisons, Table S6). Only larvae and their corresponding faeces showed no significant differences within each diet ( *B. rapa* : *p* = 0.397; *S. alba* : *p* = 0.437), supporting again the suitability of faecal microbiota as a proxy for larval microbiota. In contrast, faecal microbiota from larvae reared on different diets differed significantly (*p* = 0.001), mirroring the dietary effect observed in larval microbiota. Microbial communities differed significantly among all adult groups, including between adults exposed to clerodanoids versus unexposed individuals (Adult C+ vs. C−: *p* = 0.001, Figure S10). In addition, adult microbial communities differed significantly from those of all larval treatment groups.

### 4.3 Indicator Species Analysis Reveals Treatment‐Specific Microbial ASVs

Indicator ASV analysis revealed distinct microbial signatures across host diet, feeding status, developmental stage, and adult origin (IndVal.g ≥ 0.7; permutation *p* ≤ 0.05). Larvae fed ad libitum on *Brassica rapa* versus *Sinapis alba* were differentiated by 10 indicator ASVs (six associated with *B. rapa* ‐fed larvae, four with *S. alba* ‐fed larvae), while the comparison of larvae fed ad libitum on *B. rapa* versus starved larvae identified 15 indicator ASVs (14 associated with starved larvae, one with *B. rapa* ‐fed larvae). Among adults, 20 ASVs distinguished C+ and C− individuals (16 associated with C+ and four with C−), and 82 ASVs differentiated lab‐reared from wild‐caught adults (18 lab‐associated, 64 wild‐associated); the comparison of larvae and adults identified 15 indicator ASVs (nine adult‐associated, six larval‐associated). Full indicator species analysis results are provided in Table S7.

### 4.4 Potential Differential Metabolic Functions of Microbiota Associated With Larval Host Plant Diet, Starvation and Adult Non‐Nutritional Plant Metabolite Uptake

**FIGURE 5:**
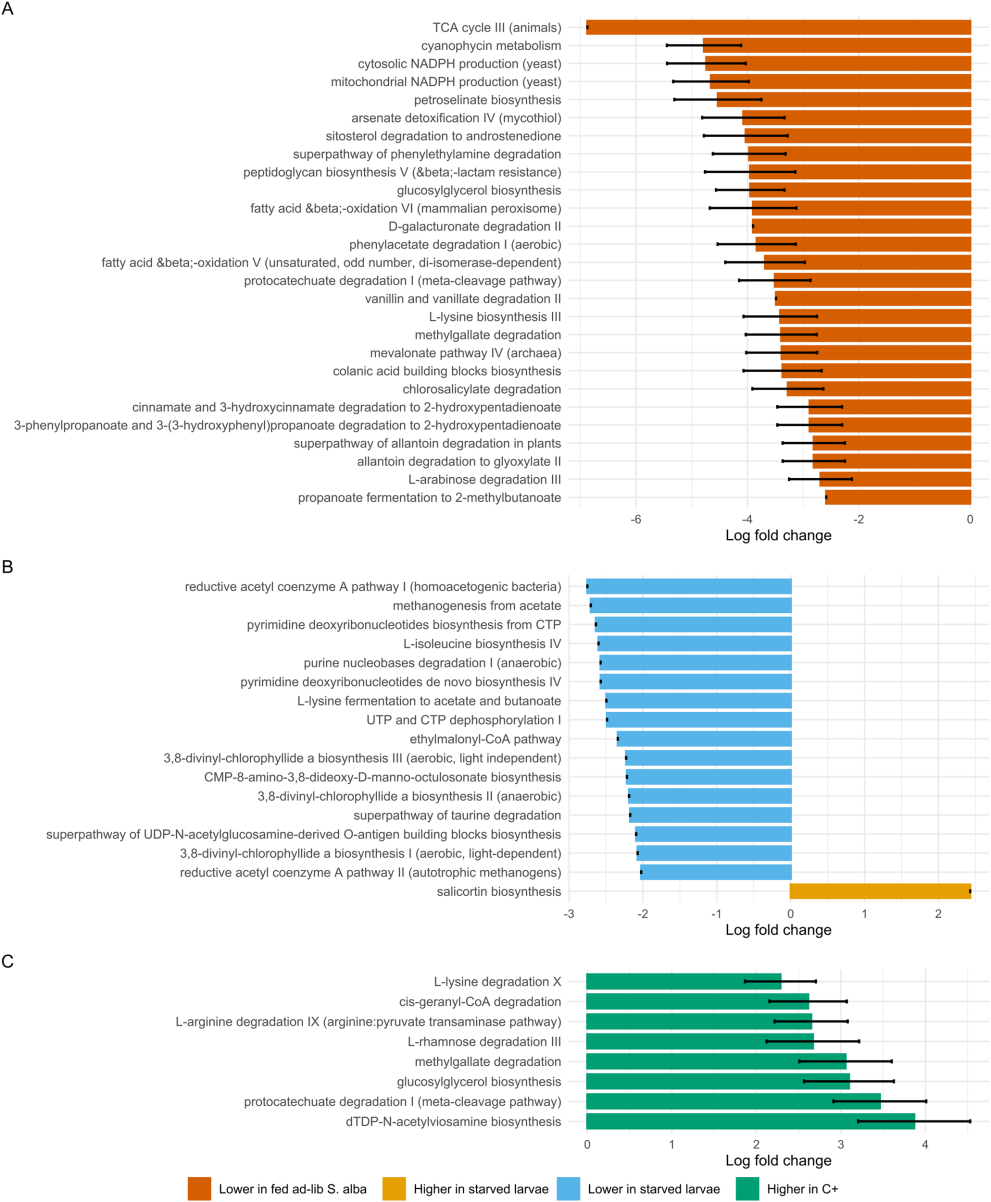
Differentially abundant predicted MetaCyc pathways in *A. rosae* (A) larvae fed ad libitum on *S. alba* versus on *B. rapa* , (B) larvae starved versus fed ad libitum on *B. rapa* , and (C) adults with (C+) versus without (C−) access to clerodanoids. Bars represent log fold changes in pathway abundance predicted by PICRUSt2 and tested with ANCOM‐BC. Only pathways with a Bonferroni‐adjusted *p* ≤ 0.001 and an absolute log fold change ≥ 2 are shown. Error bars indicate standard errors of the estimated log fold changes.

Predictive functional profiling suggested differences in the inferred microbial pathway abundances between larvae fed on *B. rapa* and those fed on *S. alba* . A total of 27 MetaCyc pathways were predicted to be significantly more abundant in *B. rapa* ‐fed larvae (Bonferroni‐adjusted *p* ≤ 0.001; |LFC| ≥ 2; Figure 5A). These included putative pathways associated with central energy metabolism (e.g., tricarboxylic acid (TCA) cycle III, fatty acid β‐oxidation, NADPH production), biosynthesis (e.g., L‐lysine biosynthesis III, glucosylglycerol biosynthesis) and degradation of (aromatic) specialised plant metabolites (e.g., methylgallate degradation, protocatechuate degradation, chlorosalicylate degradation). Several pathways involved in carbohydrate metabolism (e.g., D‐galacturonate, L‐arabinose, vanillin and vanillate degradation) and microbial detoxification (e.g., arsenate detoxification, cyanophycin metabolism) were also enriched in *B. rapa* ‐fed individuals.

Functional predictions also revealed 16 MetaCyc pathways that were significantly more abundant in *B. rapa* ‐fed larvae relative to starved individuals (Bonferroni‐adjusted *p* ≤ 0.001; |LFC| ≥ 2; Figure 5B). These included putative pathways involved in anaerobic metabolism (e.g., methanogenesis, reductive acetyl‐CoA pathway), nucleotide biosynthesis and turnover (e.g., dephosphorylation and nucleobase degradation), amino acid fermentation and surface structure biosynthesis. In contrast, only one putative pathway, salicortin biosynthesis, was enriched in starved larvae.

Functional prediction revealed eight MetaCyc pathways that were significantly more abundant in C+ adults compared to C− adults (Bonferroni‐adjusted *p* ≤ 0.001, |LFC| ≥ 2; Figure 5C). These included putative degradation pathways for amino acids and plant‐derived compounds (e.g., L‐lysine degradation X, L‐arginine degradation IX, methylgallate degradation, protocatechuate degradation I), as well as putative biosynthetic pathways associated with glucosylglycerol and dTDP‐*N*‐acetylviosamine. Notably, cis‐geranyl‐CoA degradation, involved in terpenoid breakdown, was enriched in C+ individuals, potentially reflecting microbial capacity to metabolise clerodanoids.

## 5 Discussion

Our study provides evidence that microbiota diversity and composition in *A. rosae* are shaped by ecological conditions, plant interactions, both nutritional and non‐nutritional, as well as the specific life stage. These findings highlight intraspecific variation in microbiota within an insect species and underscore the importance of ecological context when interpreting microbiome dynamics. Larval microbiota differed significantly in dependence on the larval diet, *B. rapa* versus *S. alba* , indicating a clear dietary effect on microbial composition. Such dietary correlation with microbiota aligns with findings across diverse insect taxa, where host‐plant chemistry, including nutritional profiles and plant specialised metabolites, pronouncedly influence microbial composition and abundance (Hammer and Bowers 2015; Lim et al. 2023; Liu et al. 2020). In herbivorous insects, dietary‐induced microbiome shifts can subsequently affect nutrient acquisition, detoxification capabilities and ultimately fitness (Dearing et al. 2022). Hence, diet‐dependent plasticity in microbiota may represent an adaptive mechanism by herbivores such as *A. rosae* facilitating exploitation of diverse host plants by overcoming distinct plant defences. However, further experimental validation is required to determine whether these microbial differences translate into measurable fitness benefits for the insect host. In this study, inference about plant‐associated microbial sources is limited because host plant microbiota could not be analysed due to few sequencing reads. Moreover, as eggs of all laboratory individuals were laid on *S. alba* , shared early exposure could contribute to some overlap in microbiota between diet groups, but this overlap may also result from inherited larval microbiomes, as found in many insect species (Guilhot et al. 2023; Salem et al. 2015).

In contrast, no significant differences were detected between larvae and their corresponding faeces within each diet, supporting the use of faeces as a non‐invasive proxy for larval microbiota under controlled conditions, as also shown in other insects such as *Drosophila melanogaster* (Fink et al. 2013). However, faecal samples may have come into contact with larvae and host plant material after deposition, which may have contributed to the observed correspondence between larval and faecal microbiota. Faeces from larvae reared on different host plants differed significantly, indicating that dietary effects on microbiota are detectable even in faecal samples. *S. alba* ‐fed larvae exhibited significantly higher PD than their faeces, suggesting the absence of phylogenetically distinct taxa in the faeces, potentially because some taxa, for example, those adhering to the gut lining or in non‐gut tissues, are not excreted. Moreover, faecal samples likely capture primarily gut‐associated microbiota, whereas larval samples include whole‐body‐associated microbes.

Contrary to our hypothesis, starved larvae exhibited increased Shannon diversity relative to fed larvae, without a corresponding increase in Faith's PD. This pattern indicates a change in community diversity without a corresponding increase in phylogenetic diversity. A similar pattern was observed in desert locusts after 5 days of starvation, with increased Gammaproteobacteria diversity in starved individuals, potentially enhancing colonisation resistance (Dillon et al. 2010). In contrast, black soldier flies exhibited reduced Shannon diversity after 48 h of starvation, along with shifts in dominant taxa and predicted metabolic functions (Yang et al. 2021), suggesting that starvation effects on microbiota are species‐ and context‐dependent. In *A. rosae* , we applied a 24‐h starvation period, as longer starvation is known to increase larval mortality (Paul et al. 2019), whereas a 24‐h starvation period does not significantly increase mortality (Brueggemann, Singh, and Müller 2025). The observed microbiota shift therefore likely reflects an early response to nutritional stress, while longer starvation periods may result in different outcomes.

As hypothesised, adult exposure to clerodanoids significantly influenced microbial community composition. The observed microbial shifts may result from the antimicrobial properties of clerodanoids, selectively suppressing susceptible taxa and shaping community structure accordingly (Bozov et al. 2015; Šigutová et al. 2024). Interestingly, some microbial taxa can metabolise clerodanoids, such as *Streptomyces* sp. CPCC 205437, which biotransforms the neo‐clerodane diterpenoid scutebarbatine F (Zhang et al. 2021), potentially generating novel metabolites that may influence microbial community composition. Our finding provides empirical support for the influence of non‐nutritional plant metabolites on insect microbiota, underscoring pharmacophagy as a potent factor shaping microbiome dynamics.

As expected, wild‐caught adults exhibited significantly higher microbial alpha diversity than laboratory‐reared individuals. They also displayed a significantly distinct microbiota composition compared to all other individuals, as also indicated by the many indicator ASVs differentiating them from lab‐reared adults. The distinct microbiota composition likely reflects increased microbial exposure in natural habitats, a phenomenon well‐documented across taxa, including plants and animals (Chandler et al. 2011; Malacrinò et al. 2025). Similar patterns have been reported in diverse insect species, where laboratory rearing usually results in reduced microbial richness and altered community composition relative to wild counterparts, with potential loss of environmentally acquired or functionally relevant taxa (Polidori et al. 2023; Tinker and Ottesen 2021; Waltmann et al. 2019). For example, in the Eurasian spruce bark beetle, *Ips typographus*, laboratory rearing led to reduced fungal diversity and the loss or replacement of specific microbial taxa compared to wild individuals, despite overall retention of the core microbiota (Baños‐Quintana et al. 2024).

For the wild‐caught *A. rosae* adults, their prior conditions are unknown; consequently, their intake of both primary and specialised plant metabolites such as clerodanoids, as well as their microbial exposure, likely varied among individuals. A previous study of clerodanoid contents in wild‐caught *A. rosae* found substantial variation, ranging from absence to high levels of the two major clerodanoids (Singh et al. 2024). Variation may also stem from sex differences, as both males and females were sampled. In general, elevated microbial diversity in wild insects can enhance metabolic and immune functions, supporting greater resilience to environmental stressors and confer fitness advantages (Engel and Moran 2013). Our results thus emphasise the need for caution when extrapolating laboratory findings to natural populations.

Additionally, distinct microbial communities between larvae and adults supported predictions regarding life stage specificity in microbiome composition (González‐Serrano et al. 2020; Manthey et al. 2023). Such ontogenetic shifts likely reflect ecological transitions associated with metamorphosis, including altered physiological conditions, dietary habits and ecological niches (Manthey et al. 2023). Moreover, indicator species analysis identified distinct ASVs consistently associated with larval diet, starvation, adult clerodanoid access, rearing origin and life stage, indicating that microbiota shifts across treatments are driven by specific taxa rather than uniform community‐wide changes. In contrast to the microbial community, the alpha diversity did not differ between laboratory‐reared *A. rosae* larvae and adults. In comparison, *Brithys crini* moths showed a decline in diversity from larval to adult stages, though this finding was based solely on gut microbiota (González‐Serrano et al. 2020). It remains to be tested which body parts of *A. rosae* may carry the most microbiota and may drive differences in alpha or beta diversity. Studies in other insect species suggest that microbial biomass is substantially higher within the insect body than on its surface (Hammer et al. 2015).

Our results indicate shifts in predicted microbial functional profiles in response to larval diet, nutritional stress, and adult pharmacophagy. Microbiota of *B. rapa‐*fed larvae exhibited predicted enhanced capacity for energy production, metabolising dietary substrates, synthesising essential metabolites and phytochemical processing, potentially reflecting the response to plant‐specific nutrient quality and specialised metabolites. Such reliance on microbial degradation of dietary plant compounds is well‐documented in herbivorous insects such as leaf beetles, where horizontal gene transfer and symbiosis have driven dietary adaptation (Kirsch et al. 2025), though whether similar mechanisms exist in *A. rosae* remains unknown. In contrast, starvation in *A. rosae* larvae markedly reduced microbial functional potential, with only one predicted pathway (salicortin biosynthesis) enriched, suggesting a functional bottleneck under nutrient deprivation. Furthermore, the presence of clerodanoids, which are diterpenoids, in C+ adults shifted microbial functions towards putative degradation pathways for amino acids and specialised metabolites, including terpenoid breakdown (cis‐geranyl‐CoA degradation), emphasising the role of non‐nutritional metabolites in modulating microbial communities. However, as these functional profiles were inferred from 16S rRNA gene data using PICRUSt2, they represent predicted metabolic potential rather than direct assessments of gene presence or expression or enzymatic activity. These results should therefore be interpreted with caution, and future studies employing metagenomics or metabolomics will be necessary to confirm the functional roles of the microbiota.

Taken together, our findings contribute insights into the ecological drivers shaping insect microbiota. Our results suggest that host plant species, nutritional status, non‐nutritional plant metabolites and life stage can influence microbiota diversity and composition, underscoring the context‐dependence of insect microbiota. Future work should prioritise longitudinal, field‐based investigations that explicitly link microbiota shifts to host performance and reproductive output, thereby testing whether the observed context‐dependent microbiota variation is associated with fitness differences in natural ecosystems.

## Author Contributions

**Pragya Singh:** conceptualization, investigation, writing – original draft, writing – review and editing, methodology, formal analysis, data curation, visualization, validation. **Philipp Dirksen:** conceptualisation, experimental design, writing – review and editing. **Martin Kaltenpoth:** conceptualization, methodology, funding acquisition, writing – review and editing, resources. **Caroline Müller:** resources, writing – review and editing, writing – original draft, conceptualization, methodology, funding acquisition.

## Funding

This work was supported by the Deutsche Forschungsgemeinschaft (Grant 396777467) as part of the SFB TRR 212 (NC^3^).

## Conflicts of Interest

The authors declare no conflicts of interest.

## Supporting information


**Figure S1:** emi70309‐sup‐0001‐Supinfo.docx.
**Figure S2:** emi70309‐sup‐0001‐Supinfo.docx.
**Figure S3:** emi70309‐sup‐0001‐Supinfo.docx.
**Figure S4:** emi70309‐sup‐0001‐Supinfo.docx.
**Figure S5:** emi70309‐sup‐0001‐Supinfo.docx.
**Figure S6:** emi70309‐sup‐0001‐Supinfo.docx.
**Figure S7:** emi70309‐sup‐0001‐Supinfo.docx.
**Figure S8:** emi70309‐sup‐0001‐Supinfo.docx.
**Figure S9:** emi70309‐sup‐0001‐Supinfo.docx.
**Figure S10:** emi70309‐sup‐0001‐Supinfo.docx.
**Table S1:** emi70309‐sup‐0001‐Supinfo.docx.
**Table S2:** emi70309‐sup‐0001‐Supinfo.docx.
**Table S4:** emi70309‐sup‐0001‐Supinfo.docx.
**Table S5:** emi70309‐sup‐0001‐Supinfo.docx.
**Table S6:** emi70309‐sup‐0001‐Supinfo.docx.
**Table S7:** emi70309‐sup‐0001‐Supinfo.docx.

**Table S3:** emi70309‐sup‐0003‐TableS3.zip.

## Data Availability

The datasets and R scripts are uploaded to Zenodo repository (10.5281/zenodo.19372587).
