# Supplementary material for: Ecological Drivers of Microbiota Diversity in the Pharmacophagous Turnip Sawfly
Source: Environ Microbiol. 2026 Apr 21;28:e70309. doi: 10.1111/1462-2920.70309 (PMC13097239; doi:10.1111/1462-2920.70309)
Supplement: Supplementary file 1 — Figure S1: emi70309‐sup‐0001‐Supinfo.docx. Figure S2: emi70309‐sup‐0001‐Supinfo.docx. Figure S3: emi70309‐sup‐0001‐Supinfo.docx. Figure S4: emi70309‐sup‐0001‐Supinfo.docx. Figure S5: emi70309‐sup‐0001‐Supinfo.docx. Figure S6: emi70309‐sup‐0001‐Supinfo.docx. Figure S7: emi70309‐sup‐0001‐Supinfo.docx. Figure S8: emi70309‐sup‐0001‐Supinfo.docx. Figure S9: emi70309‐sup‐0001‐Supinfo.docx. Figure S10: emi70309‐sup‐0001‐Supinfo.docx. Table S1: emi70309‐sup‐0001‐Supinfo.docx. Table S2: emi70309‐sup‐0001‐Supinfo.docx. Table S4: emi70309‐sup‐0001‐Supinfo.docx. Table S5: emi70309‐sup‐0001‐Supinfo.docx. Table S6: emi70309‐sup‐0001‐Supinfo.docx. Table S7: emi70309‐sup‐0001‐Supinfo.docx. [file EMI-28-e70309-s001.docx]

**Supplementary Information**

**S1. DNA extraction Protocol**

DNA was extracted from Athalia rosae samples (larvae, adults, and *faeces*) using the ZymoBIOMICS™ DNA Miniprep Kit (Zymo Research, Cat. No. D4300). For whole-body extractions, initial homogenisation was performed using ZR BashingBead™ Lysis Tubes with 2 mm beads (Zymo Research, Cat. No. S6003-50), followed by fine bead lysis using the 0.1 and 0.5 mm bead tubes provided in the kit. The protocol was carried out as follows:

#### ****Sample Preparation and Homogenisation****

1. Workspace, pipettes, and centrifuges were cleaned using 10% bleach followed by DNA Away (Thermo Fisher Scientific) to prevent DNA contamination.
2. One 50 mL Falcon tube containing 10% bleach and three 50 mL tubes filled with sterile Milli-Q water were prepared for the sequential decontamination of forceps and scalpels. Tools were thoroughly rinsed with water following bleach exposure to avoid sample degradation.
3. Individual insect bodies or plant tissue samples were placed into **ZR BashingBead™ Lysis Tubes (2 mm beads).**
4. Tubes and the associated tissue lyser racks were frozen at –20 °C for a minimum of 15 minutes. During homogenisation, tubes were kept on ice and spaced apart to minimize cross-contamination.
5. Samples were homogenised for 5 minutes at 30 Hz using a Qiagen TissueLyser II. If tissue lysis was incomplete, an additional 3-minute homogenisation cycle was performed.
6. Tubes were briefly centrifuged at 16,000 × g for 30 seconds at 20 °C.
7. A volume of 750 µL of **ZymoBIOMICS™ Lysis Solution** was added to each tube. A negative control consisting of lysis buffer alone was also included.
8. A second round of homogenisation was carried out for 5 minutes at 30 Hz in tissue lyser.
9. Tubes were centrifuged at ≥16,000 × g for 30 seconds.
10. The lysate (~750 µL), was transferred to **ZR BashingBead™ Lysis Tubes (0.1 & 0.5 mm beads).** Lysates were mixed thoroughly prior to transfer.
11. Tubes were vortexed for 40 minutes at maximum speed using a Vortex Genie equipped with a horizontal tube holder.

#### ****DNA Purification, Quantification, and Storage****

Prior to the DNA extraction steps, three collection tubes and two Eppendorf tubes per sample replicate were prepared and labelled accordingly (e.g., for 17 replicates, 51 collection tubes and 34 Eppendorf tubes were used). DNA-containing eluates were retained at steps 4, 11, and 13.

1. ZR BashingBead™ Lysis Tubes (0.1 & 0.5 mm beads) were centrifuged at ≥10,000 × g for 1 minute in a microcentrifuge. Samples were fully thawed prior to centrifugation.
2. Up to 400 μL of the supernatant was carefully transferred to a Zymo-Spin™ III-F Filter placed in a collection tube and centrifuged at 8,000 × g for 1 minute. Beads were excluded during transfer to avoid reducing DNA yield. The filtrate was retained, and the filter was discarded after step 5 was completed.
3. A total of 1,200 μL of ZymoBIOMICS™ DNA Binding Buffer was added to the filtrate and mixed thoroughly.
4. An 800 μL aliquot of the mixture was transferred to a Zymo-Spin™ IICR Column in a collection tube and centrifuged at 10,000 × g for 1 minute. This step was repeated with the remaining 800 μL of mixture.
5. The flow-through was discarded after each centrifugation in above step.
6. The Zymo-Spin™ IICR Column was placed into a new collection tube, and 400 μL of ZymoBIOMICS™ DNA Wash Buffer 1 was added. The sample was centrifuged at 10,000 × g for 1 minute, and the flow-through was discarded.
7. A volume of 700 μL of ZymoBIOMICS™ DNA Wash Buffer 2 was added, followed by centrifugation at 10,000 × g for 1 minute. The flow-through was discarded.
8. An additional 200 μL of DNA Wash Buffer 2 was added and centrifuged at 10,000 × g for 1 minute. The flow-through was discarded.
9. The column was transferred to a clean 1.5 mL microcentrifuge tube, and 100 μL of DNase/RNase-Free Water was added directly to the column matrix. After a 1-minute incubation at room temperature, DNA was eluted by centrifugation at 10,000 × g for 1 minute. The eluate was retained and kept on ice.
10. A Zymo-Spin™ III-HRC Filter was prepared by adding 600 μL of ZymoBIOMICS™ HRC Prep Solution into a new collection tube and centrifuging at 8,000 × g for 3 minutes.
11. The eluted DNA (~100 μL from Step 11) was transferred to the prepared HRC Filter in a clean 1.5 mL microcentrifuge tube and centrifuged at exactly 16,000 × g for 3 minutes. The filtered eluate was retained for downstream use.

The filtered DNA was used for PCR amplification and sequencing.

#### ****DNA Quantification****

DNA concentration and quality were assessed (Figure S1) using a NanoPhotometer® (Implen GmbH):

1. The NanoPhotometer surface was cleaned with sterile water before use.
2. A blank measurement was performed using 1 μL of DNase/RNase-Free Water placed at the center of the red dot on the measurement pedestal.
3. Subsequently, 1 μL of each DNA sample was loaded and measured. Samples were vortexed briefly prior to loading, if necessary.

#### ****Aliquoting for Downstream Applications****

1. For sequencing, 10 μL aliquots of each purified DNA sample were transferred to freshly labeled Eppendorf tubes.
2. For PCR amplification, 1 μL of the purified DNA was used per replicate.

#### Figures


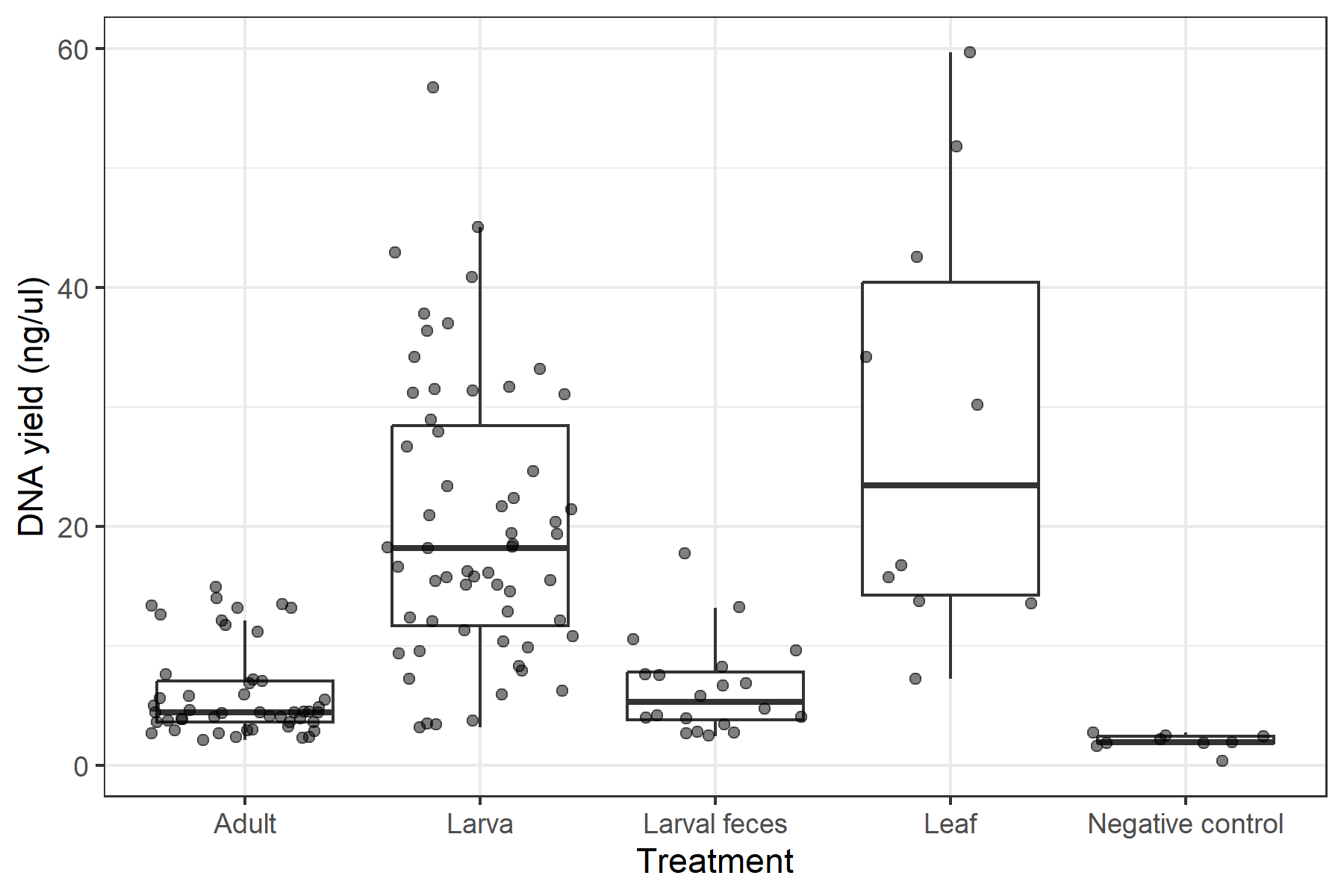


#### **Figure S1.** DNA yield (ng/µL) across different sample types used in the microbiota study. Boxplots show the distribution of extracted DNA concentrations from adult, larval, larval *faeces*, and leaf samples, alongside negative controls. Each point represents a biological replicate.


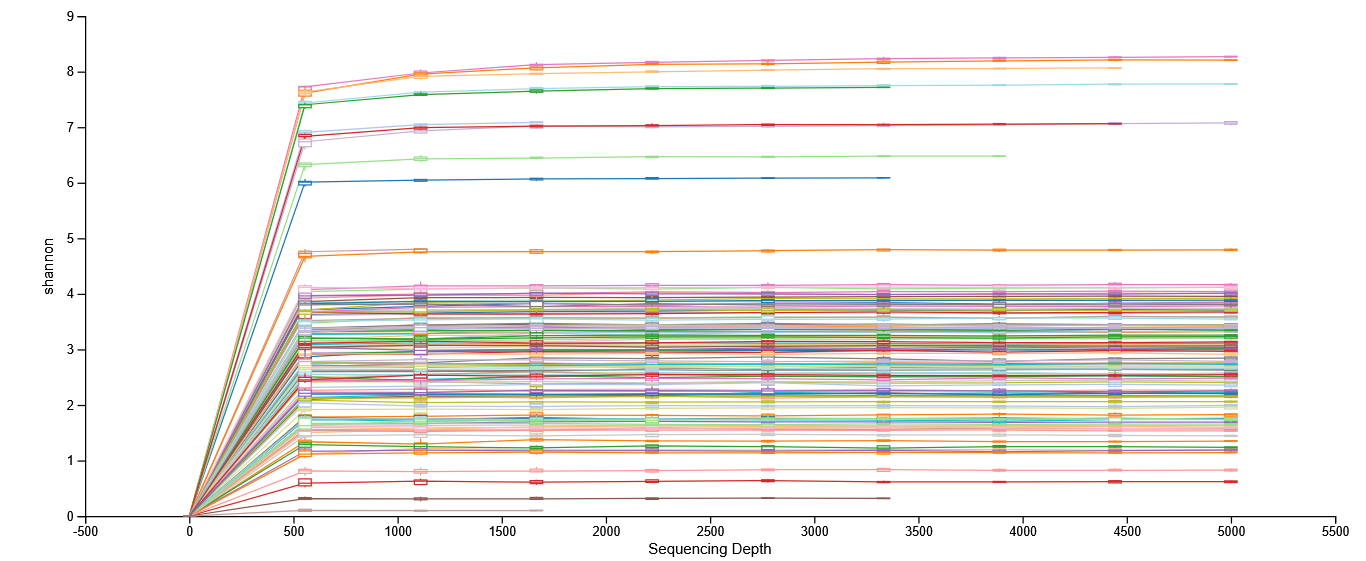


Figure S2. Rarefaction curves based on Shannon diversity index for all samples, rarefied up to a maximum sequencing depth of 5000 reads per sample. Each curve represents one sample.


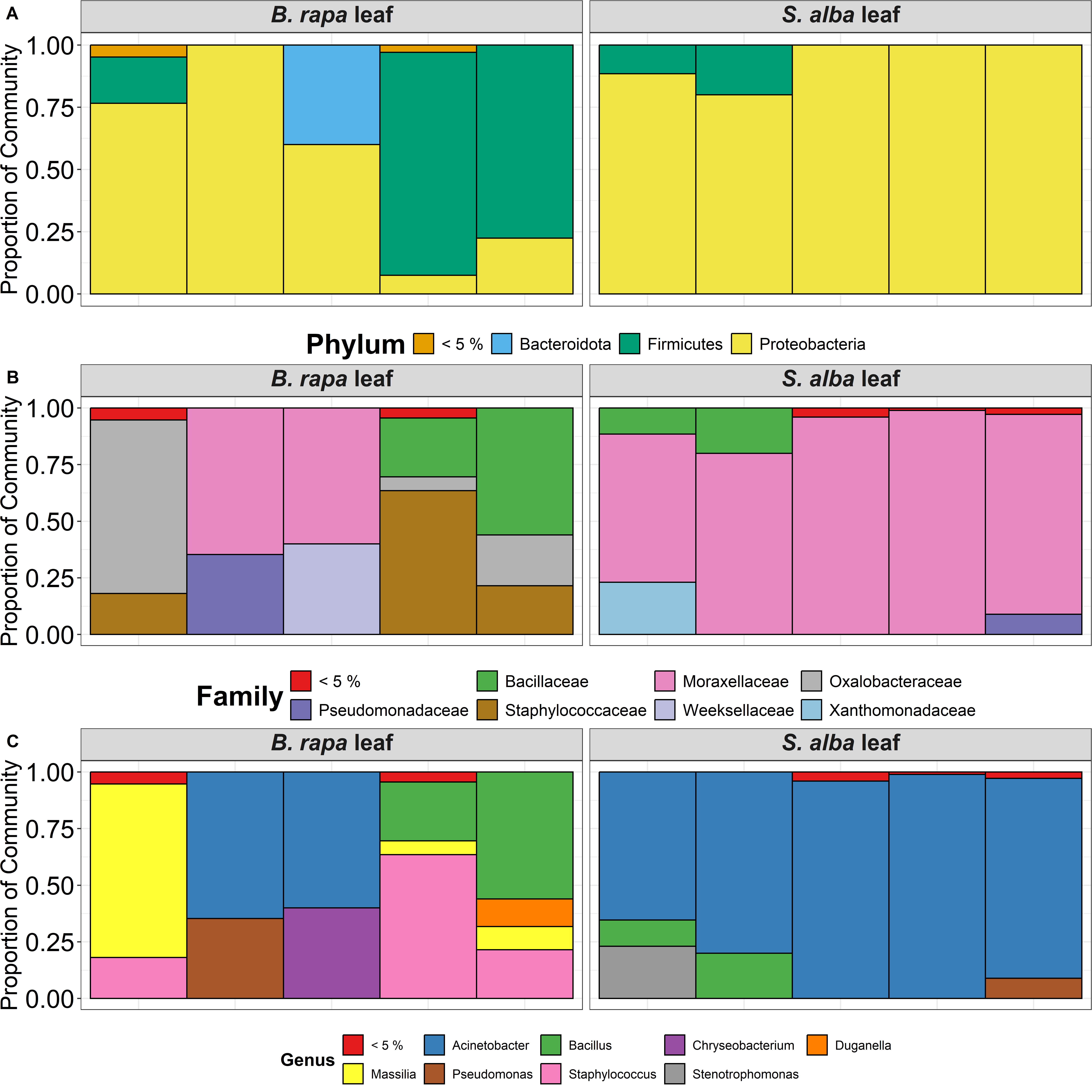


Figure S3. Microbial community composition at the (A) phylum, (B) family, and (C) genus level in leaf samples of *Brassica rapa* (n = 5) and *Sinapis alba* (n = 5). The "< 5%" category groups all taxa with a relative abundance below 5% within a given sample. These samples were excluded from the main statistical analyses, because all *B. rapa* and three *S. alba* leaf samples had fewer than 1,000 sequencing reads.


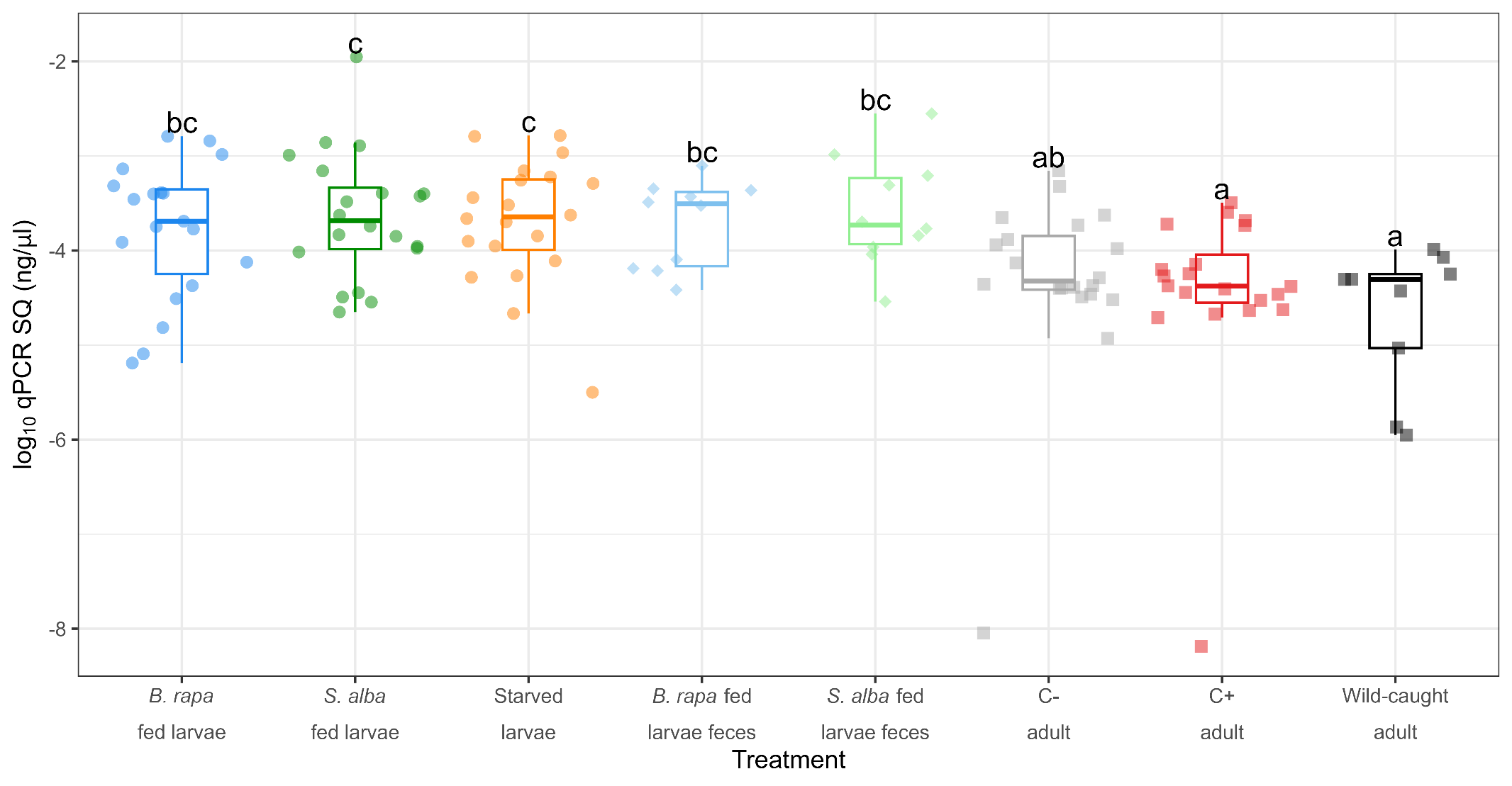


#### **Figure S4. Total bacterial abundance based on quantitative PCR (qPCR) across treatments in *Athalia rosae*. Log10-transformed starting quantity values (SQ; ng/µl) from qPCR for larval, faecal, and adult samples. Boxplots display the median, interquartile range, and individual sample points. Treatments included larvae fed ad libitum on *Brassica rapa* or *Sinapis alba*, starved larvae, faecal samples from larvae on both diets, laboratory-reared adults unexposed (C−) or exposed (C+) to clerodanoids, and wild-caught adults. Letters above boxplots indicate post hoc groupings based on Kruskal-Wallis tests followed by Dunn tests with Benjamini-Hochberg correction; groups sharing at least one letter did not differ significantly.**

Figure S5. Relative abundance of bacterial families across treatments in *Athalia rosae*, shown as stacked bar plots. Each bar represents an individual sample, and panels denote experimental groups: larvae fed ad libitum on *Brassica rapa* or *Sinapis alba*, starved larvae, faecal samples from both diets, laboratory-reared adults unexposed (C−) or exposed to clerodanoids (C+), and wild-caught adults. In wild-caught adults, the first three bars represent males, and the remaining five represent females. Only families with ≥5% relative abundance are shown.


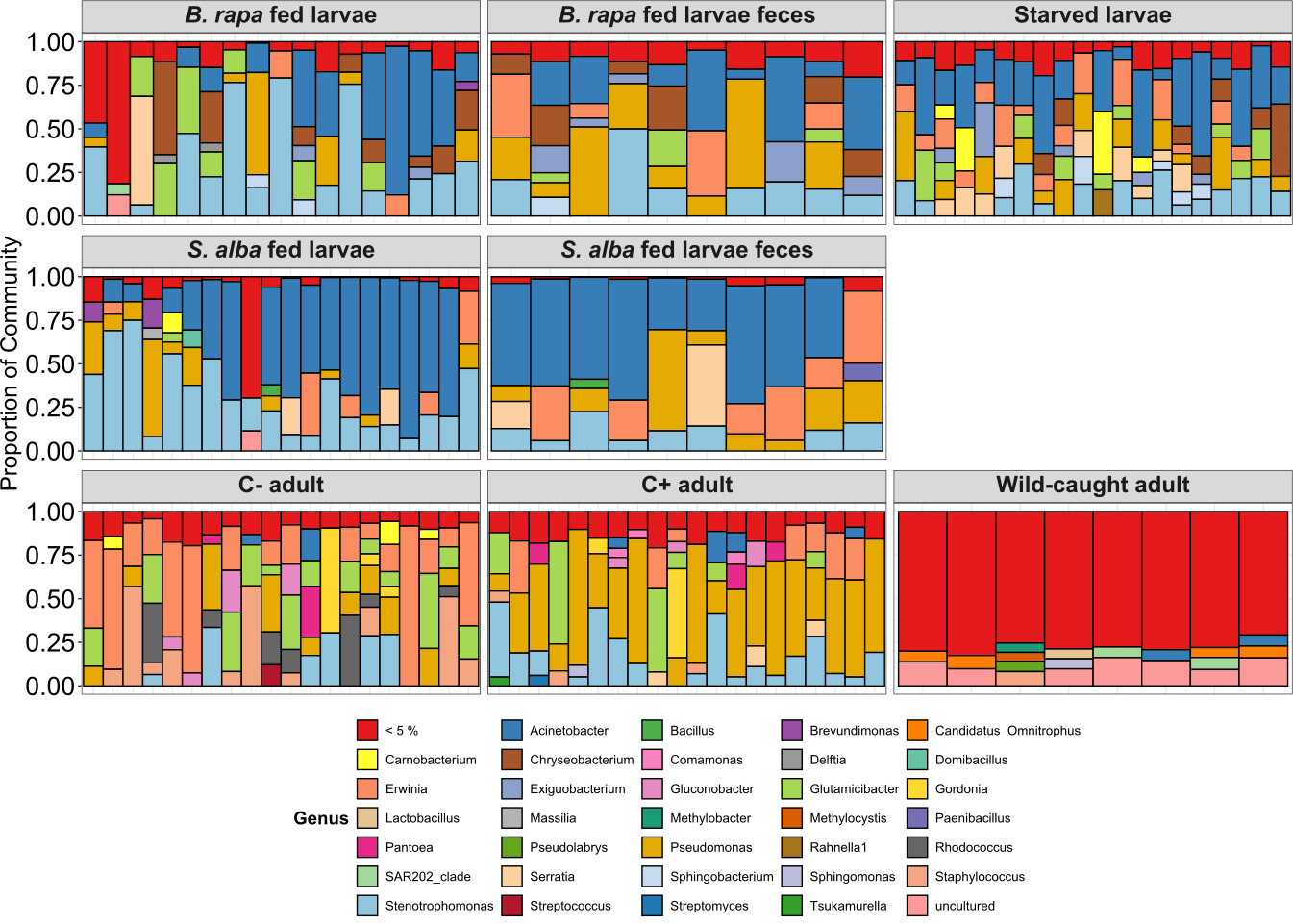


Figure S6. Relative abundance of bacterial genera across treatments in *Athalia rosae*, shown as stacked bar plots. Each bar represents an individual sample, and panels denote experimental groups: larvae fed ad libitum on *Brassica rapa* or *Sinapis alba*, starved larvae, faecal samples from both diets, laboratory-reared adults unexposed (C−) or exposed to clerodanoids (C+), and wild-caught adults. In wild-caught adults, the first three bars represent males, and the remaining five represent females. Only genera with ≥5% relative abundance are shown.


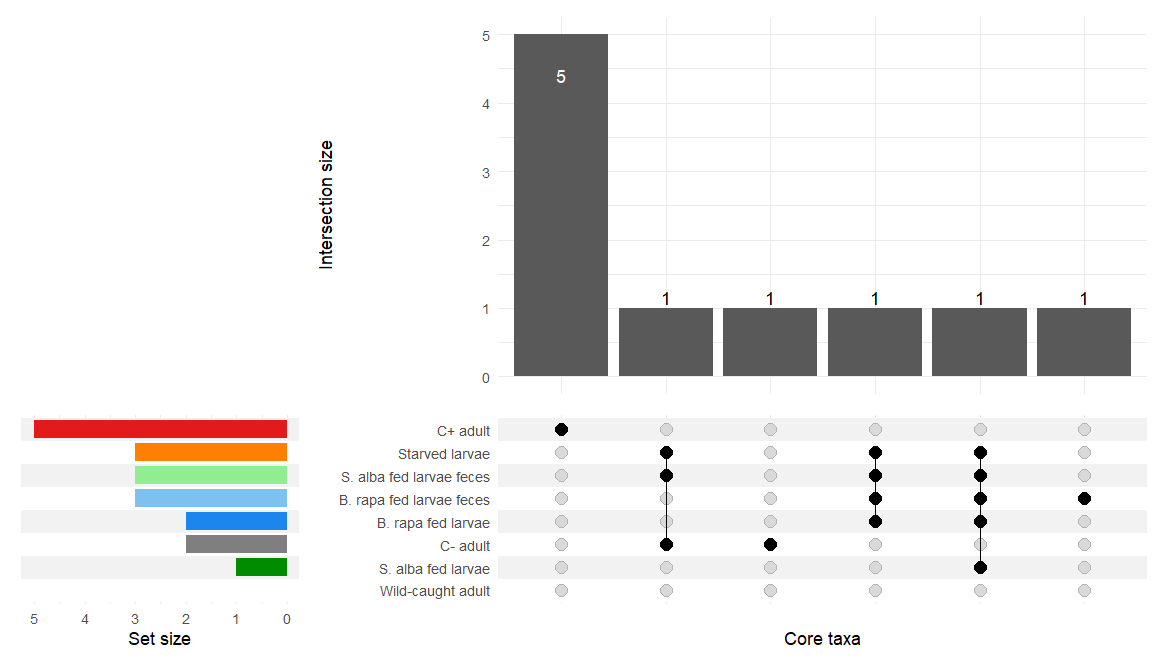


Figure S7. Overlap of treatment-specific core bacterial ASVs among *Athalia rosae* treatments (UpSet plot). Core microbiota were defined within each treatment as ASVs present in ≥70% of samples with a minimum relative abundance of ≥1%. Horizontal bars (left) show the number of core ASVs per treatment (set size). Vertical bars (top) show the number of core ASVs shared by the treatment combination indicated below (intersection size). Filled black circles connected by lines indicate treatments included in a given intersection; grey circles indicate treatments not included. Robustness of core sizes across prevalence (50%, 70%, 90%) and detection (0.1%, 0.5%, 1%, 2%) thresholds is shown in Supplementary Figure S8.


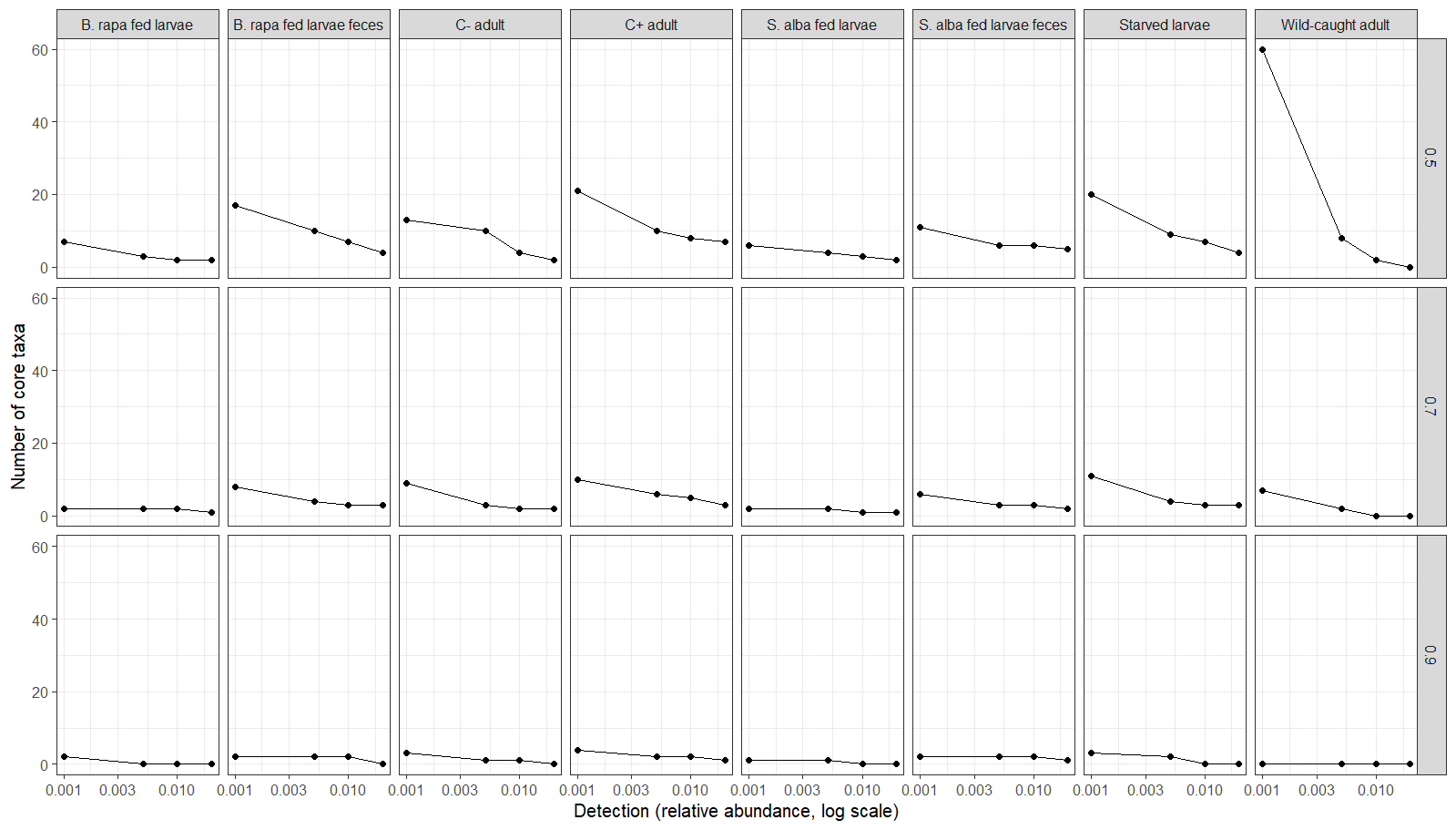


Figure S8. Number of core taxa inferred under different core-definition thresholds across experimental groups. Columns correspond to the *Athalia rosae* treatments. Rows show the prevalence threshold (0.5, 0.7, 0.9). The x-axis shows the detection threshold (minimum relative abundance; log scale) evaluated at 0.1%, 0.5%, 1%, and 2% (i.e., 0.001, 0.005, 0.01, and 0.02 as proportions).


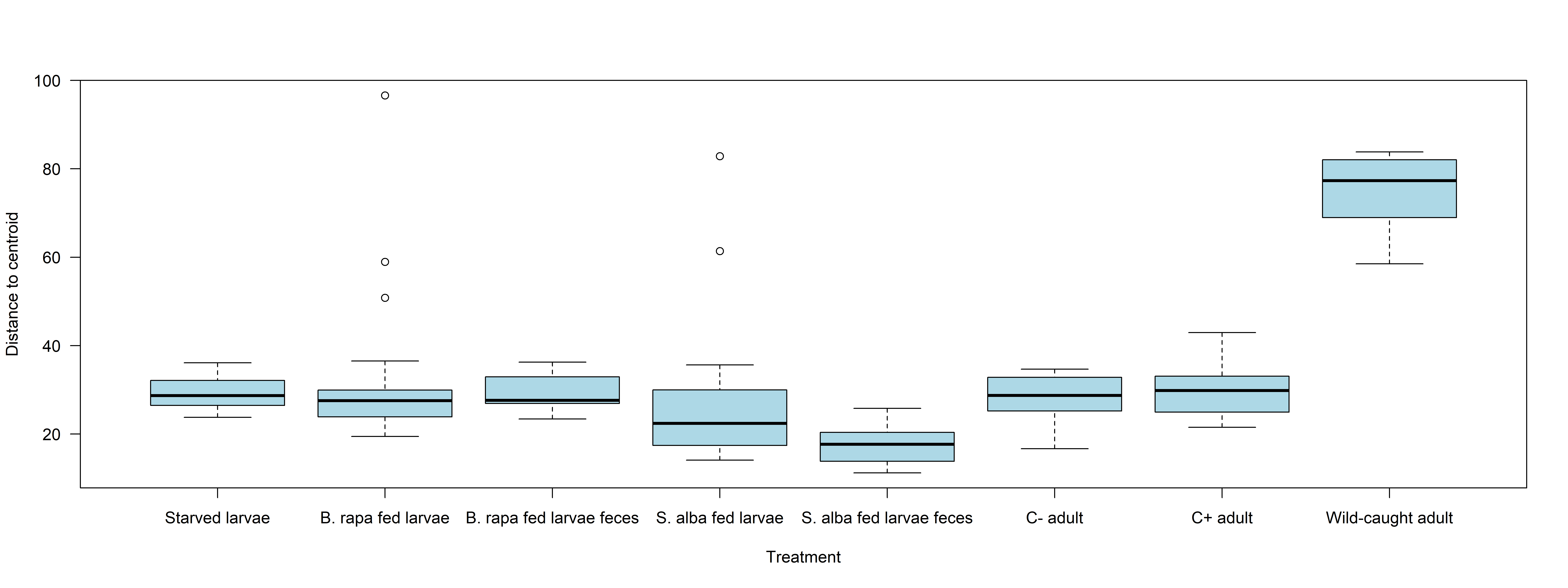


#### **Figure S9. Beta dispersion across treatment groups based on distance to centroid. Boxplots show distance to group centroid for beta diversity for each treatment group**.


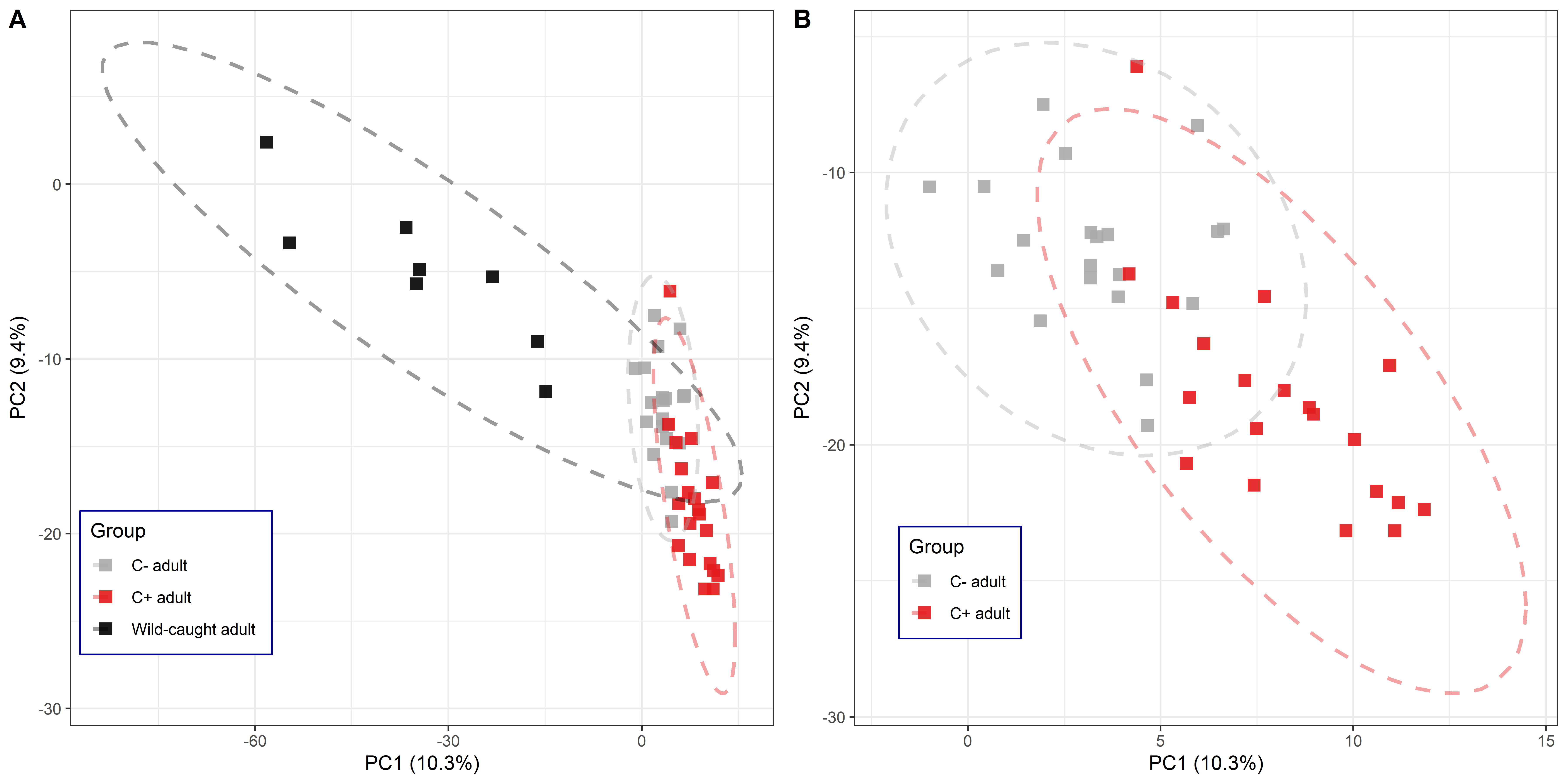


Figure S10. Principal component analysis (PCA) of CLR-transformed amplicon sequence variant (ASV) data using Aitchison distance, showing variation in microbial community composition among *Athalia rosae* adult treatments. Each point represents one sample and is coloured by treatment group. (A) Adults unexposed to clerodanoids (C−), adults exposed to clerodanoids (C+), and wild-caught adults. (B) C− and C+ adults only. Ellipses show 95% confidence intervals around group centroids. Axis labels indicate the percentage of variance explained by PC1 and PC2.

#### Tables

Table S1. **Post hoc Dunn test results for Shannon entropy (alpha diversity) of microbiota with Benjamini–Hochberg correction following Kruskal–Wallis test.** Pairwise comparisons are shown with adjusted P-values. Statistically significant comparisons (P < 0.05) are highlighted in bold.

| Comparison | Z | P.unadj | P.adj |
| --- | --- | --- | --- |
| C- Adult vs C+ Adult | -1.628 | 1.04E-01 | 0.145 |
| C- Adult vs Wild-caught adult | -4.129 | 3.65E-05 | **<0.001** |
| C+ Adult vs Wild-caught adult | -2.898 | 3.75E-03 | **0.011** |
| C- Adult vs *B. rapa fed larvae faeces* | -0.880 | 3.79E-01 | 0.424 |
| C+ Adult vs *B. rapa fed larvae faeces* | 0.449 | 6.53E-01 | 0.678 |
| Wild-caught adult vs *B. rapa fed larvae faeces* | 2.923 | 3.47E-03 | **0.011** |
| C- Adult vs *S. alba fed larvae faeces* | 1.906 | 5.66E-02 | 0.088 |
| C+ Adult vs *S. alba fed larvae faeces* | 3.236 | 1.21E-03 | **0.004** |
| Wild-caught adult vs *S. alba fed larvae faeces* | 5.198 | 2.01E-07 | **<0.001** |
| *B. rapa fed larvae faeces* vs *S. alba fed larvae faeces* | 2.413 | 1.58E-02 | **0.032** |
| C- Adult vs *B. rapa fed larvae* | 0.459 | 6.46E-01 | 0.696 |
| C+ Adult vs *B. rapa fed larvae* | 2.020 | 4.34E-02 | 0.071 |
| Wild-caught adult vs *B. rapa fed larvae* | 4.382 | 1.18E-05 | **<0.001** |
| *B. rapa fed larvae faeces* vs *B. rapa fed larvae* | 1.236 | 2.17E-01 | 0.276 |
| *S. alba fed larvae faeces* vs *B. rapa fed larvae* | -1.472 | 1.41E-01 | 0.188 |
| C- Adult vs *S. alba fed larvae* | 2.313 | 2.07E-02 | **0.039** |
| C+ Adult vs *S. alba fed larvae* | 3.941 | 8.11E-05 | **<0.001** |
| Wild-caught adult vs *S. alba fed larvae* | 5.877 | 4.17E-09 | **<0.001** |
| *B. rapa fed larvae faeces* vs *S. alba fed larvae* | 2.769 | 5.63E-03 | **0.013** |
| *S. alba fed larvae faeces* vs *S. alba fed larvae* | -0.018 | 9.86E-01 | 0.986 |
| *B. rapa fed larvae* vs *S. alba fed larvae* | 1.758 | 7.88E-02 | 0.116 |
| C- Adult vs Starved larvae | -2.510 | 1.21E-02 | **0.026** |
| C+ Adult vs Starved larvae | -0.882 | 3.78E-01 | 0.441 |
| Wild-caught adult vs Starved larvae | 2.232 | 2.56E-02 | **0.045** |
| *B. rapa fed larvae faeces* vs Starved larvae | -1.169 | 2.42E-01 | 0.295 |
| *S. alba fed larvae faeces* vs Starved larvae | -3.955 | 7.64E-05 | **<0.001** |
| *B. rapa fed larvae* vs Starved larvae | -2.865 | 4.17E-03 | **0.011** |
| *S. alba fed larvae* vs Starved larvae | -4.823 | 1.42E-06 | **<0.001** |

Table S2. **Post hoc Dunn test results for Faith’s phylogenetic diversity (alpha diversity) with Benjamini–Hochberg correction following Kruskal–Wallis test.** Adjusted P-values are shown for all pairwise comparisons. Statistically significant comparisons (P < 0.05) are highlighted in bold.

| Comparison | Z | P.unadj | P.adj |
| --- | --- | --- | --- |
| C- Adult vs C+ Adult | -2.012 | 4.42E-02 | 0.073 |
| C- Adult vs Wild-caught adult | -3.552 | 3.83E-04 | **0.001** |
| C+ Adult vs Wild-caught adult | -2.031 | 4.23E-02 | 0.074 |
| C- Adult vs *B. rapa fed larvae faeces* | 0.410 | 6.82E-01 | 0.707 |
| C+ Adult vs *B. rapa fed larvae faeces* | 2.053 | 4.01E-02 | 0.075 |
| Wild-caught adult vs *B. rapa fed larvae faeces* | 3.467 | 5.27E-04 | **0.002** |
| C- Adult vs *S. alba fed larvae faeces* | 3.952 | 7.75E-05 | **<0.001** |
| C+ Adult vs *S. alba fed larvae faeces* | 5.595 | 2.21E-08 | **<0.001** |
| Wild-caught adult vs *S. alba fed larvae faeces* | 6.359 | 2.03E-10 | **<0.001** |
| *B. rapa fed larvae faeces* vs *S. alba fed larvae faeces* | 3.068 | 2.16E-03 | **0.005** |
| C- Adult vs *B. rapa fed larvae* | -0.200 | 8.41E-01 | 0.841 |
| C+ Adult vs *B. rapa fed larvae* | 1.729 | 8.39E-02 | 0.124 |
| Wild-caught adult vs *B. rapa fed larvae* | 3.311 | 9.29E-04 | **0.003** |
| *B. rapa fed larvae faeces* vs *B. rapa fed larvae* | -0.564 | 5.73E-01 | 0.642 |
| *S. alba fed larvae faeces* vs *B. rapa fed larvae* | -4.006 | 6.17E-05 | **<0.001** |
| C- Adult vs *S. alba fed larvae* | 1.680 | 9.29E-02 | 0.130 |
| C+ Adult vs *S. alba fed larvae* | 3.692 | 2.22E-04 | **0.001** |
| Wild-caught adult vs *S. alba fed larvae* | 4.822 | 1.42E-06 | **<0.001** |
| *B. rapa fed larvae faeces* vs *S. alba fed larvae* | 0.962 | 3.36E-01 | 0.409 |
| *S. alba fed larvae faeces* vs *S. alba fed larvae* | -2.580 | 9.88E-03 | **0.020** |
| *B. rapa fed larvae* vs *S. alba fed larvae* | 1.811 | 7.02E-02 | 0.109 |
| C- Adult vs Starved larvae | 1.109 | 2.68E-01 | 0.341 |
| C+ Adult vs Starved larvae | 3.121 | 1.81E-03 | **0.005** |
| Wild-caught adult vs Starved larvae | 4.390 | 1.14E-05 | **<0.001** |
| *B. rapa fed larvae faeces* vs Starved larvae | 0.495 | 6.20E-01 | 0.668 |
| *S. alba fed larvae faeces* vs Starved larvae | -3.047 | 2.31E-03 | **0.005** |
| *B. rapa fed larvae* vs Starved larvae | 1.263 | 2.07E-01 | 0.276 |
| *S. alba fed larvae* vs Starved larvae | -0.572 | 5.68E-01 | 0.662 |

**Table S3. (A) Amplicon Sequence Variant (ASV) table including representative 16S rRNA gene sequences and taxonomic classification based on SILVA 138. (B) ASV (amplicon sequence variant) read counts per sample (Sample ID). Values are ASV read counts and reflect sequencing coverage. Files are provided separately as CSV files.**

Table S4. Core bacterial ASVs by treatment and their taxonomic annotations. Core microbiota were defined within each treatment as ASVs detected in ≥70% of samples at a minimum relative abundance ≥1%. “NA” indicates that no assignment was available at that rank. For more details of the ASV ID, see Supplementary **Table S3A.**

| Treatment | ASV ID | Kingdom | Phylum | Class | Order | Family | Genus | Species |
| --- | --- | --- | --- | --- | --- | --- | --- | --- |
| C- adult | ASV_987 | d__Bacteria | Proteobacteria | Gammaproteobacteria | Enterobacterales | Erwiniaceae | Erwinia | Erwinia_iniecta |
| C- adult | ASV_988 | d__Bacteria | Proteobacteria | Gammaproteobacteria | Enterobacterales | Erwiniaceae | Erwinia | NA |
| C+ adult | ASV_761 | d__Bacteria | Proteobacteria | Gammaproteobacteria | Pseudomonadales | Pseudomonadaceae | Pseudomonas | NA |
| C+ adult | ASV_762 | d__Bacteria | Proteobacteria | Gammaproteobacteria | Pseudomonadales | Pseudomonadaceae | Pseudomonas | NA |
| C+ adult | ASV_765 | d__Bacteria | Proteobacteria | Gammaproteobacteria | Pseudomonadales | Pseudomonadaceae | Pseudomonas | NA |
| C+ adult | ASV_825 | d__Bacteria | Proteobacteria | Gammaproteobacteria | Xanthomonadales | Xanthomonadaceae | Stenotrophomonas | Stenotrophomonas_chelatiphaga |
| C+ adult | ASV_883 | d__Bacteria | Proteobacteria | Gammaproteobacteria | Burkholderiales | Comamonadaceae | Comamonas | Comamonas_koreensis |
| *B. rapa* fed larvae faeces | ASV_495 | d__Bacteria | Bacteroidota | Bacteroidia | Flavobacteriales | Weeksellaceae | Chryseobacterium | NA |
| *B. rapa* fed larvae faeces | ASV_795 | d__Bacteria | Proteobacteria | Gammaproteobacteria | Pseudomonadales | Moraxellaceae | Acinetobacter | Acinetobacter_guillouiae |
| *B. rapa* fed larvae faeces | ASV_830 | d__Bacteria | Proteobacteria | Gammaproteobacteria | Xanthomonadales | Xanthomonadaceae | Stenotrophomonas | NA |
| *S. alba* fed larvae faeces | ASV_795 | d__Bacteria | Proteobacteria | Gammaproteobacteria | Pseudomonadales | Moraxellaceae | Acinetobacter | Acinetobacter_guillouiae |
| *S. alba* fed larvae faeces | ASV_830 | d__Bacteria | Proteobacteria | Gammaproteobacteria | Xanthomonadales | Xanthomonadaceae | Stenotrophomonas | NA |
| *S. alba* fed larvae faeces | ASV_987 | d__Bacteria | Proteobacteria | Gammaproteobacteria | Enterobacterales | Erwiniaceae | Erwinia | Erwinia_iniecta |
| *B. rapa* fed larvae | ASV_795 | d__Bacteria | Proteobacteria | Gammaproteobacteria | Pseudomonadales | Moraxellaceae | Acinetobacter | Acinetobacter_guillouiae |
| *B. rapa* fed larvae | ASV_830 | d__Bacteria | Proteobacteria | Gammaproteobacteria | Xanthomonadales | Xanthomonadaceae | Stenotrophomonas | NA |
| *S. alba* fed larvae | ASV_795 | d__Bacteria | Proteobacteria | Gammaproteobacteria | Pseudomonadales | Moraxellaceae | Acinetobacter | Acinetobacter_guillouiae |
| Starved larvae | ASV_795 | d__Bacteria | Proteobacteria | Gammaproteobacteria | Pseudomonadales | Moraxellaceae | Acinetobacter | Acinetobacter_guillouiae |
| Starved larvae | ASV_830 | d__Bacteria | Proteobacteria | Gammaproteobacteria | Xanthomonadales | Xanthomonadaceae | Stenotrophomonas | NA |
| Starved larvae | ASV_987 | d__Bacteria | Proteobacteria | Gammaproteobacteria | Enterobacterales | Erwiniaceae | Erwinia | Erwinia_iniecta |

**Table S5. Post hoc pairwise comparisons of beta dispersion among groups using Tukey’s Honest Significant Difference (HSD) test.** The test was applied to distance-to-centroid values from a betadisper object in R. P-values were adjusted using the Tukey method, which controls the family-wise error rate based on the studentised range distribution. Statistically significant comparisons (P < 0.05) are highlighted in bold.

| Comparison | diff | lwr | upr | p adj |
| --- | --- | --- | --- | --- |
| C+ Adult - C- Adult | 1.05 | -9.39 | 11.49 | 0.999 |
| Wild-caught adult - C- Adult | 46.43 | 32.62 | 60.24 | **<0.001** |
| *B. rapa fed larvae faeces* - C- Adult | 0.69 | -12.10 | 13.47 | 1 |
| *S. alba fed larvae faeces* - C- Adult | -10.70 | -23.49 | 2.09 | 0.173 |
| *B. rapa fed larvae* - C- Adult | 5.44 | -5.45 | 16.33 | 0.783 |
| *S. alba fed larvae* - C- Adult | -0.93 | -11.37 | 9.51 | 0.999 |
| Starved larvae - C- Adult | 0.74 | -9.70 | 11.18 | 0.999 |
| Wild-caught adult - C+ Adult | 45.38 | 31.57 | 59.19 | **<0.001** |
| *B. rapa fed larvae faeces* - C+ Adult | -0.36 | -13.15 | 12.42 | 1 |
| *S. alba fed larvae faeces* - C+ Adult | -11.75 | -24.54 | 1.03 | 0.096 |
| *B. rapa fed larvae* - C+ Adult | 4.39 | -6.51 | 15.28 | 0.917 |
| *S. alba fed larvae* - C+ Adult | -1.98 | -12.42 | 8.46 | 0.999 |
| Starved larvae - C+ Adult | -0.31 | -10.75 | 10.13 | 1 |
| *B. rapa fed larvae faeces* - Wild-caught adult | -45.74 | -61.41 | -30.07 | **<0.001** |
| *S. alba fed larvae faeces* - Wild-caught adult | -57.13 | -72.79 | -41.46 | **<0.001** |
| *B. rapa fed larvae* - Wild-caught adult | -40.99 | -55.15 | -26.83 | **<0.001** |
| *S. alba fed larvae* - Wild-caught adult | -47.36 | -61.17 | -33.54 | **<0.001** |
| Starved larvae - Wild-caught adult | -45.69 | -59.51 | -31.87 | **<0.001** |
| *S. alba fed larvae faeces* - *B. rapa fed larvae faeces* | -11.39 | -26.16 | 3.38 | 0.261 |
| *B. rapa fed larvae* - *B. rapa fed larvae faeces* | 4.75 | -8.41 | 17.91 | 0.952 |
| *S. alba fed larvae* - *B. rapa fed larvae faeces* | -1.62 | -14.41 | 11.17 | 0.999 |
| Starved larvae - *B. rapa fed larvae faeces* | 0.05 | -12.74 | 12.84 | 1 |
| *B. rapa fed larvae* - *S. alba fed larvae faeces* | 16.14 | 2.98 | 29.29 | **0.005** |
| *S. alba fed larvae* - *S. alba fed larvae faeces* | 9.77 | -3.02 | 22.56 | 0.272 |
| Starved larvae - *S. alba fed larvae faeces* | 11.44 | -1.35 | 24.22 | 0.115 |
| *S. alba fed larvae* - *B. rapa fed larvae* | -6.37 | -17.26 | 4.52 | 0.618 |
| Starved larvae - *B. rapa fed larvae* | -4.70 | -15.59 | 6.19 | 0.884 |
| Starved larvae - *S. alba fed larvae* | 1.67 | -8.78 | 12.11 | 0.999 |

**Table S6. Post hoc pairwise comparisons from PERMANOVA (adonis2) based on Aitchison distance for beta diversity.** Adjusted P-values (Benjamini–Hochberg correction) are shown for all pairwise comparisons. Statistically significant comparisons (P < 0.05) are highlighted in bold.

| Comparison | Df | SumsOfSqs | F.Model | R2 | p.value | p.adjusted |
| --- | --- | --- | --- | --- | --- | --- |
| C+ Adult vs C- Adult | 1 | 6777.594 | 7.478 | 0.164 | 0.001 | **0.001** |
| C+ Adult vs *B. rapa fed larvae faeces* | 1 | 8078.754 | 8.543 | 0.234 | 0.001 | **0.001** |
| C+ Adult vs *S. alba fed larvae* | 1 | 10328.888 | 10.335 | 0.214 | 0.001 | **0.001** |
| C+ Adult vs *B. rapa fed larvae* | 1 | 9386.640 | 7.668 | 0.180 | 0.001 | **0.001** |
| C+ Adult vs Wild-caught adult | 1 | 14216.845 | 5.851 | 0.184 | 0.001 | **0.001** |
| C+ Adult vs Starved larvae | 1 | 11892.395 | 12.875 | 0.253 | 0.001 | **0.001** |
| C+ Adult vs *S. alba fed larvae faeces* | 1 | 6391.104 | 8.441 | 0.232 | 0.001 | **0.001** |
| C- Adult vs *B. rapa fed larvae faeces* | 1 | 6564.646 | 7.294 | 0.207 | 0.001 | **0.001** |
| C- Adult vs *S. alba fed larvae* | 1 | 7759.295 | 8.035 | 0.175 | 0.001 | **0.001** |
| C- Adult vs *B. rapa fed larvae* | 1 | 7148.274 | 6.019 | 0.147 | 0.001 | **0.001** |
| C- Adult vs Wild-caught adult | 1 | 12069.581 | 5.070 | 0.163 | 0.001 | **0.001** |
| C- Adult vs Starved larvae | 1 | 9851.685 | 11.069 | 0.226 | 0.001 | **0.001** |
| C- Adult vs *S. alba fed larvae faeces* | 1 | 4840.994 | 6.804 | 0.195 | 0.001 | **0.001** |
| *B. rapa fed larvae faeces* vs *S. alba fed larvae* | 1 | 3695.164 | 3.601 | 0.114 | 0.001 | **0.001** |
| *B. rapa fed larvae faeces* vs *B. rapa fed larvae* | 1 | 1385.889 | 1.031 | 0.040 | 0.383 | 0.397 |
| *B. rapa fed larvae faeces* vs Wild-caught adult | 1 | 10436.016 | 3.096 | 0.162 | 0.001 | **0.001** |
| *B. rapa fed larvae faeces* vs Starved larvae | 1 | 1400.663 | 1.517 | 0.051 | 0.024 | **0.025** |
| *B. rapa fed larvae faeces* vs *S. alba fed larvae faeces* | 1 | 2518.640 | 3.791 | 0.174 | 0.001 | **0.001** |
| *S. alba fed larvae* vs *B. rapa fed larvae* | 1 | 3218.292 | 2.498 | 0.067 | 0.001 | **0.001** |
| *S. alba fed larvae* vs Wild-caught adult | 1 | 9160.666 | 3.640 | 0.123 | 0.001 | **0.001** |
| *S. alba fed larvae* vs Starved larvae | 1 | 6513.874 | 6.626 | 0.148 | 0.001 | **0.001** |
| *S. alba fed larvae* vs *S. alba fed larvae faeces* | 1 | 839.617 | 1.002 | 0.035 | 0.437 | 0.437 |
| *B. rapa fed larvae* vs Wild-caught adult | 1 | 9327.080 | 3.052 | 0.117 | 0.001 | **0.001** |
| *B. rapa fed larvae* vs Starved larvae | 1 | 3199.259 | 2.652 | 0.070 | 0.001 | **0.001** |
| *B. rapa fed larvae* vs *S. alba fed larvae faeces* | 1 | 2430.595 | 2.145 | 0.079 | 0.001 | **0.001** |
| Wild-caught adult vs Starved larvae | 1 | 14370.452 | 5.973 | 0.187 | 0.001 | **0.001** |
| Wild-caught adult vs *S. alba fed larvae faeces* | 1 | 8215.845 | 2.702 | 0.144 | 0.001 | **0.001** |
| Starved larvae vs *S. alba fed larvae faeces* | 1 | 3748.360 | 5.100 | 0.154 | 0.001 | **0.001** |

Table S7. Indicator species (ASV) analysis (IndVal.g) for *A. rosae*. For each comparison, ASVs significantly associated with a treatment are shown. Binary columns indicate group association (1 = associated with that group; 0 = not associated). stat gives the IndVal.g value and p.value the permutation test P-value. The comparisons were (a) larvae fed ad libitum on *Sinapis alba* vs *Brassica rapa*; (b) larvae fed ad libitum on *B. rapa* vs starved larvae; (c) lab-reared adults C+ vs C−; (d) lab-reared adults (C+ and C− combined) vs wild-caught adults, and (e) adults (C+, C- and wild-caught) vs larvae (*S. alba* fed, *B. rapa* fed and starved).

(a) larvae fed ad libitum on *Sinapis alba* vs *Brassica rapa*

| ASV | *B. rapa* fed larvae | *S. alba* fed larvae | stat | p.value |
| --- | --- | --- | --- | --- |
| ASV_495 | 1 | 0 | 0.804 | 0.001 |
| ASV_1507 | 1 | 0 | 0.804 | 0.001 |
| ASV_788 | 1 | 0 | 0.789 | 0.012 |
| ASV_873 | 1 | 0 | 0.767 | 0.001 |
| ASV_1322 | 1 | 0 | 0.728 | 0.002 |
| ASV_1542 | 1 | 0 | 0.726 | 0.001 |
| ASV_767 | 1 | 0 | 0.686 | 0.001 |
| ASV_1323 | 1 | 0 | 0.650 | 0.015 |
| ASV_515 | 1 | 0 | 0.642 | 0.002 |
| ASV_883 | 1 | 0 | 0.642 | 0.004 |
| ASV_768 | 1 | 0 | 0.594 | 0.009 |
| ASV_793 | 1 | 0 | 0.594 | 0.006 |
| ASV_804 | 1 | 0 | 0.594 | 0.008 |
| ASV_512 | 1 | 0 | 0.594 | 0.003 |
| ASV_493 | 1 | 0 | 0.593 | 0.004 |
| ASV_882 | 1 | 0 | 0.542 | 0.013 |
| ASV_1006 | 1 | 0 | 0.536 | 0.048 |
| ASV_511 | 1 | 0 | 0.485 | 0.032 |
| ASV_517 | 1 | 0 | 0.485 | 0.041 |
| ASV_667 | 1 | 0 | 0.485 | 0.034 |
| ASV_799 | 1 | 0 | 0.485 | 0.035 |
| ASV_802 | 1 | 0 | 0.485 | 0.035 |
| ASV_827 | 0 | 1 | 0.819 | 0.003 |
| ASV_828 | 0 | 1 | 0.744 | 0.012 |
| ASV_826 | 0 | 1 | 0.707 | 0.022 |
| ASV_829 | 0 | 1 | 0.705 | 0.014 |
| ASV_1536 | 0 | 1 | 0.698 | 0.017 |
| ASV_765 | 0 | 1 | 0.637 | 0.009 |
| ASV_998 | 0 | 1 | 0.592 | 0.01 |
| ASV_1001 | 0 | 1 | 0.547 | 0.03 |
| ASV_972 | 0 | 1 | 0.500 | 0.049 |

(b) larvae fed ad libitum on *B. rapa* vs starved larvae

| ASV | *B. rapa* fed larvae | Starved larvae | stat | p.value |
| --- | --- | --- | --- | --- |
| ASV_1507 | 1 | 0 | 0.792 | 0.001 |
| ASV_656 | 1 | 0 | 0.542 | 0.014 |
| ASV_1067 | 1 | 0 | 0.485 | 0.036 |
| ASV_788 | 0 | 1 | 0.898 | 0.001 |
| ASV_987 | 0 | 1 | 0.873 | 0.003 |
| ASV_988 | 0 | 1 | 0.822 | 0.001 |
| ASV_768 | 0 | 1 | 0.810 | 0.01 |
| ASV_1542 | 0 | 1 | 0.806 | 0.023 |
| ASV_804 | 0 | 1 | 0.792 | 0.003 |
| ASV_1018 | 0 | 1 | 0.787 | 0.007 |
| ASV_511 | 0 | 1 | 0.778 | 0.002 |
| ASV_805 | 0 | 1 | 0.772 | 0.001 |
| ASV_1017 | 0 | 1 | 0.762 | 0.007 |
| ASV_515 | 0 | 1 | 0.753 | 0.009 |
| ASV_1339 | 0 | 1 | 0.709 | 0.009 |
| ASV_1522 | 0 | 1 | 0.707 | 0.002 |
| ASV_807 | 0 | 1 | 0.703 | 0.002 |
| ASV_1020 | 0 | 1 | 0.698 | 0.005 |
| ASV_518 | 0 | 1 | 0.696 | 0.003 |
| ASV_790 | 0 | 1 | 0.695 | 0.019 |
| ASV_791 | 0 | 1 | 0.655 | 0.033 |
| ASV_802 | 0 | 1 | 0.652 | 0.049 |
| ASV_799 | 0 | 1 | 0.651 | 0.047 |
| ASV_808 | 0 | 1 | 0.632 | 0.005 |
| ASV_998 | 0 | 1 | 0.632 | 0.007 |
| ASV_1001 | 0 | 1 | 0.632 | 0.014 |

(c) lab-reared adults C+ vs C−

| ASV | C+ adults | C- adults | stat | p.value |
| --- | --- | --- | --- | --- |
| ASV_761 | 1 | 0 | 0.970 | 0.001 |
| ASV_726 | 1 | 0 | 0.949 | 0.001 |
| ASV_825 | 1 | 0 | 0.949 | 0.001 |
| ASV_883 | 1 | 0 | 0.949 | 0.001 |
| ASV_1280 | 1 | 0 | 0.936 | 0.001 |
| ASV_765 | 1 | 0 | 0.932 | 0.001 |
| ASV_779 | 1 | 0 | 0.922 | 0.001 |
| ASV_774 | 1 | 0 | 0.913 | 0.001 |
| ASV_762 | 1 | 0 | 0.894 | 0.001 |
| ASV_498 | 1 | 0 | 0.837 | 0.001 |
| ASV_804 | 1 | 0 | 0.837 | 0.001 |
| ASV_857 | 1 | 0 | 0.806 | 0.001 |
| ASV_489 | 1 | 0 | 0.775 | 0.001 |
| ASV_500 | 1 | 0 | 0.775 | 0.001 |
| ASV_482 | 1 | 0 | 0.707 | 0.001 |
| ASV_525 | 1 | 0 | 0.707 | 0.001 |
| ASV_671 | 1 | 0 | 0.671 | 0.003 |
| ASV_716 | 1 | 0 | 0.632 | 0.005 |
| ASV_720 | 1 | 0 | 0.632 | 0.003 |
| ASV_521 | 1 | 0 | 0.592 | 0.006 |
| ASV_696 | 1 | 0 | 0.592 | 0.011 |
| ASV_524 | 1 | 0 | 0.548 | 0.025 |
| ASV_870 | 1 | 0 | 0.548 | 0.017 |
| ASV_1515 | 0 | 1 | 0.910 | 0.002 |
| ASV_987 | 0 | 1 | 0.895 | 0.001 |
| ASV_988 | 0 | 1 | 0.849 | 0.001 |
| ASV_1323 | 0 | 1 | 0.750 | 0.023 |
| ASV_1514 | 0 | 1 | 0.632 | 0.006 |
| ASV_772 | 0 | 1 | 0.632 | 0.008 |
| ASV_1282 | 0 | 1 | 0.583 | 0.029 |
| ASV_466 | 0 | 1 | 0.565 | 0.042 |

(d) lab-reared adults (C+ and C− combined) vs wild-caught adults

| ASV | lab-reared adults (C+ and C− combined) | wild-caught adults | stat | p.value |
| --- | --- | --- | --- | --- |
| ASV_637 | 1 | 0 | 0.922 | 0.003 |
| ASV_987 | 1 | 0 | 0.894 | 0.001 |
| ASV_658 | 1 | 0 | 0.891 | 0.003 |
| ASV_777 | 1 | 0 | 0.837 | 0.014 |
| ASV_989 | 1 | 0 | 0.827 | 0.02 |
| ASV_1515 | 1 | 0 | 0.819 | 0.036 |
| ASV_1281 | 1 | 0 | 0.806 | 0.03 |
| ASV_988 | 1 | 0 | 0.791 | 0.015 |
| ASV_990 | 1 | 0 | 0.791 | 0.024 |
| ASV_1284 | 1 | 0 | 0.791 | 0.033 |
| ASV_830 | 1 | 0 | 0.771 | 0.026 |
| ASV_1322 | 1 | 0 | 0.758 | 0.023 |
| ASV_1323 | 1 | 0 | 0.756 | 0.035 |
| ASV_761 | 1 | 0 | 0.742 | 0.032 |
| ASV_765 | 1 | 0 | 0.742 | 0.027 |
| ASV_693 | 1 | 0 | 0.725 | 0.035 |
| ASV_993 | 1 | 0 | 0.725 | 0.046 |
| ASV_1017 | 1 | 0 | 0.707 | 0.041 |
| ASV_296 | 0 | 1 | 0.935 | 0.001 |
| ASV_750 | 0 | 1 | 0.935 | 0.001 |
| ASV_862 | 0 | 1 | 0.866 | 0.001 |
| ASV_1094 | 0 | 1 | 0.866 | 0.001 |
| ASV_1254 | 0 | 1 | 0.866 | 0.001 |
| ASV_1369 | 0 | 1 | 0.866 | 0.001 |
| ASV_1398 | 0 | 1 | 0.866 | 0.001 |
| ASV_69 | 0 | 1 | 0.791 | 0.001 |
| ASV_86 | 0 | 1 | 0.791 | 0.001 |
| ASV_152 | 0 | 1 | 0.791 | 0.001 |
| ASV_419 | 0 | 1 | 0.791 | 0.001 |
| ASV_701 | 0 | 1 | 0.791 | 0.001 |
| ASV_749 | 0 | 1 | 0.791 | 0.001 |
| ASV_796 | 0 | 1 | 0.791 | 0.001 |
| ASV_849 | 0 | 1 | 0.791 | 0.001 |
| ASV_952 | 0 | 1 | 0.791 | 0.001 |
| ASV_1041 | 0 | 1 | 0.791 | 0.001 |
| ASV_1053 | 0 | 1 | 0.791 | 0.001 |
| ASV_1055 | 0 | 1 | 0.791 | 0.001 |
| ASV_1201 | 0 | 1 | 0.791 | 0.001 |
| ASV_1260 | 0 | 1 | 0.791 | 0.001 |
| ASV_1352 | 0 | 1 | 0.791 | 0.001 |
| ASV_1381 | 0 | 1 | 0.791 | 0.001 |
| ASV_656 | 0 | 1 | 0.777 | 0.001 |
| ASV_12 | 0 | 1 | 0.707 | 0.001 |
| ASV_29 | 0 | 1 | 0.707 | 0.001 |
| ASV_38 | 0 | 1 | 0.707 | 0.003 |
| ASV_71 | 0 | 1 | 0.707 | 0.002 |
| ASV_78 | 0 | 1 | 0.707 | 0.001 |
| ASV_109 | 0 | 1 | 0.707 | 0.002 |
| ASV_144 | 0 | 1 | 0.707 | 0.001 |
| ASV_180 | 0 | 1 | 0.707 | 0.001 |
| ASV_210 | 0 | 1 | 0.707 | 0.001 |
| ASV_221 | 0 | 1 | 0.707 | 0.002 |
| ASV_254 | 0 | 1 | 0.707 | 0.001 |
| ASV_299 | 0 | 1 | 0.707 | 0.001 |
| ASV_353 | 0 | 1 | 0.707 | 0.001 |
| ASV_381 | 0 | 1 | 0.707 | 0.001 |
| ASV_440 | 0 | 1 | 0.707 | 0.001 |
| ASV_567 | 0 | 1 | 0.707 | 0.001 |
| ASV_568 | 0 | 1 | 0.707 | 0.002 |
| ASV_615 | 0 | 1 | 0.707 | 0.001 |
| ASV_813 | 0 | 1 | 0.707 | 0.002 |
| ASV_815 | 0 | 1 | 0.707 | 0.001 |
| ASV_922 | 0 | 1 | 0.707 | 0.003 |
| ASV_927 | 0 | 1 | 0.707 | 0.002 |
| ASV_936 | 0 | 1 | 0.707 | 0.001 |
| ASV_943 | 0 | 1 | 0.707 | 0.001 |
| ASV_947 | 0 | 1 | 0.707 | 0.003 |
| ASV_957 | 0 | 1 | 0.707 | 0.001 |
| ASV_974 | 0 | 1 | 0.707 | 0.001 |
| ASV_1078 | 0 | 1 | 0.707 | 0.001 |
| ASV_1126 | 0 | 1 | 0.707 | 0.001 |
| ASV_1142 | 0 | 1 | 0.707 | 0.002 |
| ASV_1144 | 0 | 1 | 0.707 | 0.002 |
| ASV_1145 | 0 | 1 | 0.707 | 0.001 |
| ASV_1204 | 0 | 1 | 0.707 | 0.001 |
| ASV_1218 | 0 | 1 | 0.707 | 0.001 |
| ASV_1226 | 0 | 1 | 0.707 | 0.001 |
| ASV_1367 | 0 | 1 | 0.707 | 0.002 |
| ASV_1403 | 0 | 1 | 0.707 | 0.001 |
| ASV_1405 | 0 | 1 | 0.707 | 0.001 |
| ASV_1409 | 0 | 1 | 0.707 | 0.001 |
| ASV_1484 | 0 | 1 | 0.707 | 0.003 |
| ASV_788 | 0 | 1 | 0.691 | 0.004 |
| ASV_1 | 0 | 1 | 0.612 | 0.006 |
| ASV_3 | 0 | 1 | 0.612 | 0.008 |
| ASV_16 | 0 | 1 | 0.612 | 0.003 |
| ASV_18 | 0 | 1 | 0.612 | 0.003 |
| ASV_51 | 0 | 1 | 0.612 | 0.006 |
| ASV_52 | 0 | 1 | 0.612 | 0.003 |
| ASV_67 | 0 | 1 | 0.612 | 0.004 |
| ASV_76 | 0 | 1 | 0.612 | 0.006 |
| ASV_79 | 0 | 1 | 0.612 | 0.007 |
| ASV_96 | 0 | 1 | 0.612 | 0.009 |
| ASV_103 | 0 | 1 | 0.612 | 0.005 |
| ASV_113 | 0 | 1 | 0.612 | 0.003 |
| ASV_117 | 0 | 1 | 0.612 | 0.004 |
| ASV_120 | 0 | 1 | 0.612 | 0.008 |
| ASV_122 | 0 | 1 | 0.612 | 0.006 |
| ASV_147 | 0 | 1 | 0.612 | 0.008 |
| ASV_178 | 0 | 1 | 0.612 | 0.006 |
| ASV_184 | 0 | 1 | 0.612 | 0.008 |
| ASV_198 | 0 | 1 | 0.612 | 0.008 |
| ASV_205 | 0 | 1 | 0.612 | 0.006 |
| ASV_214 | 0 | 1 | 0.612 | 0.008 |
| ASV_228 | 0 | 1 | 0.612 | 0.008 |
| ASV_240 | 0 | 1 | 0.612 | 0.006 |
| ASV_316 | 0 | 1 | 0.612 | 0.006 |
| ASV_333 | 0 | 1 | 0.612 | 0.003 |
| ASV_337 | 0 | 1 | 0.612 | 0.003 |
| ASV_340 | 0 | 1 | 0.612 | 0.003 |
| ASV_350 | 0 | 1 | 0.612 | 0.003 |
| ASV_351 | 0 | 1 | 0.612 | 0.003 |
| ASV_358 | 0 | 1 | 0.612 | 0.003 |
| ASV_361 | 0 | 1 | 0.612 | 0.006 |
| ASV_366 | 0 | 1 | 0.612 | 0.006 |
| ASV_370 | 0 | 1 | 0.612 | 0.003 |
| ASV_372 | 0 | 1 | 0.612 | 0.005 |
| ASV_376 | 0 | 1 | 0.612 | 0.004 |
| ASV_382 | 0 | 1 | 0.612 | 0.007 |
| ASV_384 | 0 | 1 | 0.612 | 0.004 |
| ASV_388 | 0 | 1 | 0.612 | 0.008 |
| ASV_391 | 0 | 1 | 0.612 | 0.005 |
| ASV_403 | 0 | 1 | 0.612 | 0.003 |
| ASV_409 | 0 | 1 | 0.612 | 0.005 |
| ASV_429 | 0 | 1 | 0.612 | 0.003 |
| ASV_430 | 0 | 1 | 0.612 | 0.004 |
| ASV_433 | 0 | 1 | 0.612 | 0.005 |
| ASV_434 | 0 | 1 | 0.612 | 0.005 |
| ASV_460 | 0 | 1 | 0.612 | 0.004 |
| ASV_463 | 0 | 1 | 0.612 | 0.009 |
| ASV_561 | 0 | 1 | 0.612 | 0.008 |
| ASV_562 | 0 | 1 | 0.612 | 0.005 |
| ASV_575 | 0 | 1 | 0.612 | 0.006 |
| ASV_577 | 0 | 1 | 0.612 | 0.005 |
| ASV_585 | 0 | 1 | 0.612 | 0.003 |
| ASV_596 | 0 | 1 | 0.612 | 0.004 |
| ASV_618 | 0 | 1 | 0.612 | 0.004 |
| ASV_623 | 0 | 1 | 0.612 | 0.008 |
| ASV_645 | 0 | 1 | 0.612 | 0.005 |
| ASV_646 | 0 | 1 | 0.612 | 0.006 |
| ASV_648 | 0 | 1 | 0.612 | 0.005 |
| ASV_683 | 0 | 1 | 0.612 | 0.003 |
| ASV_684 | 0 | 1 | 0.612 | 0.003 |
| ASV_717 | 0 | 1 | 0.612 | 0.002 |
| ASV_746 | 0 | 1 | 0.612 | 0.005 |
| ASV_751 | 0 | 1 | 0.612 | 0.003 |
| ASV_785 | 0 | 1 | 0.612 | 0.005 |
| ASV_822 | 0 | 1 | 0.612 | 0.005 |
| ASV_845 | 0 | 1 | 0.612 | 0.009 |
| ASV_868 | 0 | 1 | 0.612 | 0.005 |
| ASV_869 | 0 | 1 | 0.612 | 0.008 |
| ASV_871 | 0 | 1 | 0.612 | 0.008 |
| ASV_874 | 0 | 1 | 0.612 | 0.003 |
| ASV_875 | 0 | 1 | 0.612 | 0.004 |
| ASV_891 | 0 | 1 | 0.612 | 0.005 |
| ASV_940 | 0 | 1 | 0.612 | 0.003 |
| ASV_945 | 0 | 1 | 0.612 | 0.008 |
| ASV_962 | 0 | 1 | 0.612 | 0.003 |
| ASV_966 | 0 | 1 | 0.612 | 0.006 |
| ASV_972 | 0 | 1 | 0.612 | 0.004 |
| ASV_973 | 0 | 1 | 0.612 | 0.003 |
| ASV_975 | 0 | 1 | 0.612 | 0.005 |
| ASV_1057 | 0 | 1 | 0.612 | 0.009 |
| ASV_1059 | 0 | 1 | 0.612 | 0.004 |
| ASV_1067 | 0 | 1 | 0.612 | 0.006 |
| ASV_1068 | 0 | 1 | 0.612 | 0.003 |
| ASV_1080 | 0 | 1 | 0.612 | 0.004 |
| ASV_1085 | 0 | 1 | 0.612 | 0.002 |
| ASV_1093 | 0 | 1 | 0.612 | 0.003 |
| ASV_1107 | 0 | 1 | 0.612 | 0.008 |
| ASV_1112 | 0 | 1 | 0.612 | 0.003 |
| ASV_1119 | 0 | 1 | 0.612 | 0.005 |
| ASV_1140 | 0 | 1 | 0.612 | 0.006 |
| ASV_1151 | 0 | 1 | 0.612 | 0.003 |
| ASV_1156 | 0 | 1 | 0.612 | 0.006 |
| ASV_1167 | 0 | 1 | 0.612 | 0.006 |
| ASV_1177 | 0 | 1 | 0.612 | 0.008 |
| ASV_1207 | 0 | 1 | 0.612 | 0.006 |
| ASV_1210 | 0 | 1 | 0.612 | 0.004 |
| ASV_1215 | 0 | 1 | 0.612 | 0.006 |
| ASV_1219 | 0 | 1 | 0.612 | 0.008 |
| ASV_1237 | 0 | 1 | 0.612 | 0.004 |
| ASV_1249 | 0 | 1 | 0.612 | 0.003 |
| ASV_1255 | 0 | 1 | 0.612 | 0.005 |
| ASV_1272 | 0 | 1 | 0.612 | 0.004 |
| ASV_1354 | 0 | 1 | 0.612 | 0.005 |
| ASV_1361 | 0 | 1 | 0.612 | 0.002 |
| ASV_1365 | 0 | 1 | 0.612 | 0.006 |
| ASV_1380 | 0 | 1 | 0.612 | 0.007 |
| ASV_1385 | 0 | 1 | 0.612 | 0.008 |
| ASV_1391 | 0 | 1 | 0.612 | 0.004 |
| ASV_1402 | 0 | 1 | 0.612 | 0.008 |
| ASV_1410 | 0 | 1 | 0.612 | 0.004 |
| ASV_1424 | 0 | 1 | 0.612 | 0.006 |
| ASV_1426 | 0 | 1 | 0.612 | 0.008 |
| ASV_1434 | 0 | 1 | 0.612 | 0.008 |
| ASV_1483 | 0 | 1 | 0.612 | 0.003 |
| ASV_1486 | 0 | 1 | 0.612 | 0.008 |
| ASV_1489 | 0 | 1 | 0.612 | 0.008 |
| ASV_133 | 0 | 1 | 0.607 | 0.008 |
| ASV_651 | 0 | 1 | 0.573 | 0.02 |

(e) adults (C+, C- and wild-caught) vs larvae (*S. alba* fed, *B. rapa* fed and starved)

| ASV | Adults (C+, C- and wild-caught) | larvae (*S. alba* fed, *B. rapa* fed and starved) | stat | p.value |
| --- | --- | --- | --- | --- |
| ASV_658 | 1 | 0 | 0.887 | 0.001 |
| ASV_637 | 1 | 0 | 0.841 | 0.001 |
| ASV_989 | 1 | 0 | 0.775 | 0.001 |
| ASV_1515 | 1 | 0 | 0.764 | 0.001 |
| ASV_1281 | 1 | 0 | 0.763 | 0.001 |
| ASV_1280 | 1 | 0 | 0.760 | 0.001 |
| ASV_535 | 1 | 0 | 0.732 | 0.001 |
| ASV_990 | 1 | 0 | 0.722 | 0.001 |
| ASV_1284 | 1 | 0 | 0.722 | 0.001 |
| ASV_783 | 1 | 0 | 0.685 | 0.001 |
| ASV_761 | 1 | 0 | 0.677 | 0.001 |
| ASV_993 | 1 | 0 | 0.661 | 0.001 |
| ASV_1302 | 1 | 0 | 0.661 | 0.001 |
| ASV_765 | 1 | 0 | 0.655 | 0.001 |
| ASV_726 | 1 | 0 | 0.645 | 0.001 |
| ASV_1509 | 1 | 0 | 0.628 | 0.001 |
| ASV_825 | 1 | 0 | 0.612 | 0.001 |
| ASV_693 | 1 | 0 | 0.611 | 0.001 |
| ASV_762 | 1 | 0 | 0.577 | 0.001 |
| ASV_883 | 1 | 0 | 0.564 | 0.018 |
| ASV_1553 | 1 | 0 | 0.559 | 0.001 |
| ASV_779 | 1 | 0 | 0.557 | 0.005 |
| ASV_498 | 1 | 0 | 0.540 | 0.001 |
| ASV_1340 | 1 | 0 | 0.521 | 0.001 |
| ASV_471 | 1 | 0 | 0.520 | 0.001 |
| ASV_857 | 1 | 0 | 0.520 | 0.001 |
| ASV_489 | 1 | 0 | 0.500 | 0.001 |
| ASV_500 | 1 | 0 | 0.500 | 0.001 |
| ASV_727 | 1 | 0 | 0.500 | 0.001 |
| ASV_1464 | 1 | 0 | 0.499 | 0.007 |
| ASV_916 | 1 | 0 | 0.497 | 0.001 |
| ASV_1277 | 1 | 0 | 0.479 | 0.001 |
| ASV_1288 | 1 | 0 | 0.479 | 0.001 |
| ASV_1496 | 1 | 0 | 0.479 | 0.001 |
| ASV_466 | 1 | 0 | 0.456 | 0.002 |
| ASV_482 | 1 | 0 | 0.456 | 0.001 |
| ASV_525 | 1 | 0 | 0.456 | 0.001 |
| ASV_1278 | 1 | 0 | 0.456 | 0.002 |
| ASV_671 | 1 | 0 | 0.433 | 0.001 |
| ASV_772 | 1 | 0 | 0.433 | 0.002 |
| ASV_1282 | 1 | 0 | 0.433 | 0.001 |
| ASV_1529 | 1 | 0 | 0.432 | 0.004 |
| ASV_696 | 1 | 0 | 0.408 | 0.002 |
| ASV_716 | 1 | 0 | 0.408 | 0.001 |
| ASV_720 | 1 | 0 | 0.408 | 0.001 |
| ASV_1514 | 1 | 0 | 0.408 | 0.001 |
| ASV_521 | 1 | 0 | 0.382 | 0.002 |
| ASV_524 | 1 | 0 | 0.354 | 0.006 |
| ASV_870 | 1 | 0 | 0.354 | 0.007 |
| ASV_651 | 1 | 0 | 0.347 | 0.019 |
| ASV_1094 | 1 | 0 | 0.346 | 0.021 |
| ASV_1369 | 1 | 0 | 0.342 | 0.026 |
| ASV_1398 | 1 | 0 | 0.341 | 0.021 |
| ASV_69 | 1 | 0 | 0.323 | 0.021 |
| ASV_144 | 1 | 0 | 0.289 | 0.037 |
| ASV_568 | 1 | 0 | 0.289 | 0.042 |
| ASV_813 | 1 | 0 | 0.289 | 0.035 |
| ASV_943 | 1 | 0 | 0.289 | 0.043 |
| ASV_1226 | 1 | 0 | 0.289 | 0.048 |
| ASV_1405 | 1 | 0 | 0.289 | 0.038 |
| ASV_1484 | 1 | 0 | 0.289 | 0.031 |
| ASV_795 | 0 | 1 | 0.991 | 0.001 |
| ASV_788 | 0 | 1 | 0.834 | 0.001 |
| ASV_771 | 0 | 1 | 0.796 | 0.001 |
| ASV_830 | 0 | 1 | 0.791 | 0.001 |
| ASV_712 | 0 | 1 | 0.725 | 0.001 |
| ASV_495 | 0 | 1 | 0.723 | 0.001 |
| ASV_827 | 0 | 1 | 0.675 | 0.001 |
| ASV_1542 | 0 | 1 | 0.670 | 0.001 |
| ASV_1018 | 0 | 1 | 0.657 | 0.001 |
| ASV_873 | 0 | 1 | 0.633 | 0.001 |
| ASV_828 | 0 | 1 | 0.606 | 0.002 |
| ASV_1536 | 0 | 1 | 0.606 | 0.001 |
| ASV_1017 | 0 | 1 | 0.606 | 0.041 |
| ASV_826 | 0 | 1 | 0.592 | 0.001 |
| ASV_829 | 0 | 1 | 0.562 | 0.001 |
| ASV_515 | 0 | 1 | 0.553 | 0.001 |
| ASV_768 | 0 | 1 | 0.553 | 0.003 |
| ASV_1560 | 0 | 1 | 0.545 | 0.001 |
| ASV_1020 | 0 | 1 | 0.541 | 0.001 |
| ASV_493 | 0 | 1 | 0.530 | 0.001 |
| ASV_767 | 0 | 1 | 0.530 | 0.002 |
| ASV_511 | 0 | 1 | 0.529 | 0.003 |
| ASV_799 | 0 | 1 | 0.513 | 0.001 |
| ASV_998 | 0 | 1 | 0.513 | 0.001 |
| ASV_1001 | 0 | 1 | 0.513 | 0.002 |
| ASV_832 | 0 | 1 | 0.508 | 0.007 |
| ASV_780 | 0 | 1 | 0.506 | 0.003 |
| ASV_512 | 0 | 1 | 0.494 | 0.002 |
| ASV_715 | 0 | 1 | 0.490 | 0.003 |
| ASV_882 | 0 | 1 | 0.489 | 0.003 |
| ASV_802 | 0 | 1 | 0.489 | 0.005 |
| ASV_805 | 0 | 1 | 0.478 | 0.001 |
| ASV_1507 | 0 | 1 | 0.478 | 0.001 |
| ASV_1339 | 0 | 1 | 0.476 | 0.003 |
| ASV_517 | 0 | 1 | 0.459 | 0.001 |
| ASV_518 | 0 | 1 | 0.439 | 0.004 |
| ASV_791 | 0 | 1 | 0.439 | 0.004 |
| ASV_807 | 0 | 1 | 0.439 | 0.001 |
| ASV_1546 | 0 | 1 | 0.419 | 0.004 |
| ASV_908 | 0 | 1 | 0.397 | 0.009 |
| ASV_1006 | 0 | 1 | 0.395 | 0.039 |
| ASV_793 | 0 | 1 | 0.375 | 0.014 |
| ASV_808 | 0 | 1 | 0.375 | 0.007 |
| ASV_659 | 0 | 1 | 0.350 | 0.025 |
| ASV_497 | 0 | 1 | 0.324 | 0.018 |
| ASV_896 | 0 | 1 | 0.324 | 0.031 |
